# Supplementary material for: Prediction of Thylakoid Lipid Binding Sites on Photosystem II
Source: Biophys J. 2017 Dec 19;113(12):2669–81. doi: 10.1016/j.bpj.2017.09.039 (PMC5770566; doi:10.1016/j.bpj.2017.09.039)
Supplement: Document S1. Supporting Materials and Methods, Figs. S1 and S2, and Tables S1–S4 [file mmc1.pdf]

Biophysical Journal, Volume *113*

Supplemental Information

Prediction of Thylakoid Lipid Binding Sites on Photosystem II

Floris J. Van Eerden, Manuel N. Melo, Pim W.J.M. Frederix, and Siewert J. Marrink

Supporting Material

Prediction of Thylakoid Lipid Binding Sites on Photosystem II

Floris J. Van Eerden[1], Manuel N. Melo[1,2], Pim W.J.M. Frederix[1], Siewert J. Marrink[1]

[1] Groningen Biomolecular Sciences and Biotechnology Institute & Zernike Institute for Advanced Materials, University of Groningen, Nijenborgh 4, 9747 AG Groningen, The Netherlands. [2] Instituto de Tecnologia Química e Biológica António Xavier, Universidade Nova de Lisboa, Av. da República, 2780-157 Oeiras, Portugal.

A description of every MGDG and SQDG binding site, two tables with details of the membrane-exposed binding sites (Table S1) and co-crystallized binding sites (Table S2), two tables with details on the co-crystallized lipids (Table S3, S4), and two figures with lipid residence time histograms (Fig S1) and lipid occupancies (Fig S2) for each binding site.

Detailed description of MGDG binding sites

Here we turn to a more detailed analysis of the 9 individual binding sites of MGDG (MS1-5 on the stromal side, and ML1-4 on the lumenal side), see Figures 3, 6, S1, S2 and Table S1.

MS1 is one of the two MGDG binding sites that are located at the dimer interface. The MS1 site tightly binds lipids with residence times reaching more than 80 µs, which is due to a strong coordination by CP47-CHL26, see Figure 6A. The MS1 site mostly binds a single lipid, and is occupied by MGDG 80% of the time.

MS2 is located close to the second PLQ exchange channel (1). This site is an intermediate strong binder, with residence times up to 48 µs. The binding site is partly made up by residues from the Cyt b 559β loop, which folds back into the membrane. The PsbX helix contributes to the site, but in one of the monomers this helix moves away during the simulation. A heme is also part of the binding site, but does not seem to interact very strongly with the lipids. MS2 also binds mostly a single lipid, and is occupied 89% of the time.

MS3 is located at the short side of PSII, between CP43 and PsbZ. It does not bind lipids very strongly, with a maximum residence time of 14 µs. However, it can bind multiple MGDGs at the same time. On average, two lipids are bound, and the occupancy level is correspondingly high (94% of the time at least one MGDG lipid is found at this site). CP43-CHL40 is the main residue contributing to the MS3 binding site.

MS4 is also located at the long side of PSII and is composed out of two loops of the CP43 subunit. It does not bind lipids very strongly, with a maximum residence time of 13 μs. Most lipids bind and unbind on a sub-microsecond time scale. Similar to MS3, the site can bind multiple MGDG lipids at the same time (on average 1.8 lipids), and has an occupancy level of 90%. In particular threonine is enriched in this binding site.

MS5 is located close to the dimer interface. It is an intermediate strong binder, with residence times up to 28 μs. The occupancy level is 76%, with typically a single MGDG lipid bound. MS5 is composed out of residues from D1 and PsbI.

ML1 is the second MGDG binding site that is located right at the dimer interface. Like the stromal site MS1, it is a strong binder with residence times up to 81 μs. In fact, most of the time the site has two or three MGDG lipids bound, see Figure 6B. The overall occupancy is 84%. CP47-CHL17 has a strong coordinating function. In the dimer interface residues from both monomers interact simultaneously with the individual lipids, thereby probably contributing to the long lipid residence times in this site.

The ML2 site is located at the CP47 subunit, relatively close to the dimer interface. It is not a strong binder, probably because the site is very exposed and does not shield the lipids from the bulk membrane. Nevertheless, the site is occupied 90% of the time, and binds on average 1.5 MGDG lipids.

ML3 is located close to PLQ exchange channel II (1). It binds on average 2.1 lipids and is occupied for 96% of the simulation. There is one odd lipid that resides for more than 70 μs at the site. At a certain point during the simulation, the residues D2-THR102 and Cyt b 559 α -PHE47 move away from each other, which results in the opening of a kind of 'cove' in the protein. Subsequently a lipid enters into the cove after which the two amino acids move back to their original positions and enclose the lipid. The 'cove' opens again for a few short moments during the simulation, but in the end the lipid escapes by diving with its headgroup underneath the enclosing residues.

ML4 is located next to CP43 and is composed entirely out of residues from CP43. It is very exposed to the membrane and it binds lipids only very weakly (sub-microsecond time scale). Like ML2 and ML3, the binding site can bind multiple lipids at the same time, resulting in a high occupancy level (93%).

Detailed description of SQDG binding sites

Here we present a more detailed analysis of the 4 individual binding sites of SQDG (SS1-3 and SL1), see Figures 3, S1, S2 and Table S1.

The stromal SQDG site SS1 is located in the corner between CP43 and PsbZ. The site is not a strong binder, and is occupied only about 75% of the time, mostly by a single lipid. The site contains two positively charged residues, Lys37 and Arg135. The site also contains a CHL, but the CHL does not appear to coordinate the SQDG lipids very strongly.

SS2 is located next to the CP43 subunit. It does bind lipids somewhat stronger than site SS1, with residence times up to 7 μs and an occupancy level of 86%. The CP43-Lys156 residue is likely the main residue responsible for attracting SQDG lipids to this site.

SS3 is located between CP43 and PsbI, it is formed by an elongated stretch of residues. Both residues from CP43 and PsbI contribute to the site. The site has an occupancy of 93%. One lipid has a residence time up to 84 μs. The oleoyl tail of this lipid penetrates into the protein between CP43 and PsbI. Here it is surrounded by and possibly interacts with CHL 33 and with the tails of CHL 10, 28, 32 and 34. These interactions might stabilize the lipid resulting in its high residence time.

At the lumenal side there is one large SQDG binding site (SL1) that can simultaneously bind two or three lipids, see Figure 6C. It is located between subunits Cyt b 559 α , Cyt b 559 β , PsbJ, PsbK and ycf12 at the same spot as the entry of the PLQ/plastoquinol (PLQol) exchange channel, which grants excess to the PLQ exchange cavity. The SL1 site is a strong binder, with residence times of 10s of μs. The occupancy level is 98%. The strong binding may be explained by the presence of Mg^{2+} and Ca^{2+} ions at the binding site. Interestingly, one SQDG lipid enters and subsequently leaves the PLQ exchange cavity during the simulation.

Supplementary Tables S1-S4

Table S1: Membrane lipid bindings sites in PSII with their composition, averaged over both monomers.[a]

Binding site	Amino acids composing the binding site
	MGDG
	Stromal
MS1	CP47-LEU122, CP47-PHE123, CP47-ARG124, CP47-ASP125, CP47-ALA132, CP47-LEU133, CP47-ASP134, CP47-MET138, CP47-HIS142, CP47-CHL26, CP47-CHL27, PsbH-LEU7, PsbH-LEU11
MS2	D2-TRP21, D2-LEU22, D2-LYS23, D2-ARG24, D2-ASP25, D2-ARG26, D2-PHE27, D2-VAL28, D2-PHE29, D2-VAL30, Cyt b 559 α -TYR19, Cyt b 559 α -ILE22, Cyt b 559 α -HIS23, Cyt b 559 α -THR26, Cyt b 559 β -SER3, Cyt b 559 β -ASN4, Cyt b 559 β -VAL18, Cyt b 559 β -HEM641
MS3	CP43-CHL40
MS4	CP43-ASN155, CP43-LYS156, CP43-THR 158, CP43-THR159, CP43-HIS251, CP43-ILE252, CP43-LEU253 CP43-THR254, CP43-THR255, CP43-PRO256, CP43-PHE257
MS5	D1-ASN12, D1-TRP14, D1-GLU15, D1-CYS18, D1-TRP32, PsbI-GLY22, PsbI-PHE23, PsbI-SER25, PsbI-GLY26, PsbI-ASP27, PsbI-ALA29, PsbI-ARG30
	Lumenal
ML1[b]	D1-SER101*, D1-LEU102*, D1-ASP103*, CP47-TRP75, CP47-ASP87, CP47-PRO88, CP47-GLY89, CP47-PHE90, CP47-TRP91, CP47-CHL17, PsbO-LYS69*
ML2	CP47-ALA182, CP47-PRO183, CP47-GLU184, CP47-TRP185, CP47-GLY186
ML3	D2-ASP100, D2-PHE101, D2-THR102, D2-ARG103, Cyt b 559 α -PHE47
ML4	CP43-ASN201, CP43-PRO202, CP43-THR203, CP43-LEU204, CP43-ASP205
	SQDG
	Stromal
SS1	CP43-TRP33, CP43-LYS37, CP43-TYR131, CP43-ARG135, CP43-CHL40 PsbZ-TYR27, PsbZ-TRP33
SS2	CP43-SER144, CP43-SER145, CP43-PHE146, CP43-TYR149, CP43-LYS156, CP43-THR159
SS3	CP43-THR254, CP43-THR255, CP43-PRO256, CP43-PHE257, CP43-GLY258, CP43-TRP259, CP43-ARG261, CP43-ARG262, PsbI-SER25, PsbI-GLY26, PsbI-LYS35
	Lumenal
SL1	Cyt b 559 α -SER39, Cyt b 559 α -THR40, Cyt b 559 α -GLY41, Cyt b 559 β -GLN41, Cyt b 559 β -PHE42, Cyt b 559 β -ILE43, Cyt b 559 β -GLN44, Cyt b 559 β -ARG45, Cyt b 559 β -Ca^{2+}796, PsbJ-TYR30, PsbJ-ALA34, PsbJ-GLY35, PsbJ-LEU36, PsbJ-MG^{2+}771, ycf12-VAL18

[a] Binding sites are labelled as follow: first letter is the first letter of the lipid head group name (M for MGDG, S for SQDG), second letter indicates location, S for stromal and L for lumenal, followed by a sequential number. Positively charged residues are in blue and negatively charged residues in red.

*[b] The ML1 site is composed out of residues from both monomers, here an * behind a residue indicates that the residue is not part of the monomer to which the binding site is assigned, but belongs to the other monomer.*

Table S2: Co-crystalized lipids and their mobility.[a]

Nr	Headgroup	Mobility	Remarks
			Lumenal lipids
5	DGDG	None	
6	DGDG	None	
11	PG	Limited	
12	PG	Large	
13	SQDG	Limited	
14	SQDG	None	
21	SQDG	Limited	
22	SQDG	Large	An extra SQDG lipid enters the PLQ exchange cavity
23	SQDG	Limited	
24	SQDG	Large	An extra SQDG lipid enters the PLQ exchange cavity
27	SQDG	Limited	
28	SQDG	Limited	
29	SQDG	Limited	
30	SQDG	Limited	
45	SQDG	None	
46	SQDG	Escape	Escapes after diffusion of PsbX
47	PG	Limited	
48	PG	Limited	
49	PG	None	
50	PG	None	
51	PG	Limited	
52	PG	None	
55	PG	Limited	
56	PG	Limited	
59	PG	Limited	
60	PG	None	
61	MGDG	Escape	
62	MGDG	Escape	
			Stromal lipids
1	DGDG	None	
2	DGDG	None	
3	DGDG	None	
4	DGDG	None	
7	DGDG	Exchange	Lipid 7 & 20 are basically at the same spot, they both escape, but only one DGDG lipid returns
8	DGDG	Escape	
9	DGDG	Escape	No lipid density visible, so therefore not shown in Figure 3
10	DGDG	Escape	No lipid density visible, so therefore not shown in Figure 3
17	DGDG	None	
18	DGDG	Escape	The escape is a result of the movement of PsbX, which moves away and opens up the binding site. No lipid density visible in Figure 3.
19	DGDG	Escape	
20	DGDG	Exchange	Lipid 7 & 20 are basically at the same spot, they both escape, but only one DGDG lipid replaces them
25	MGDG	Limited	
26	MGDG	Limited	
31	MGDG	None	
32	MGDG	None	
33	DGDG	None	
34	DGDG	None	
35	DGDG	Exchange	Leaves the PLQ exchange cavity and gets replaced by another DGDG lipid
36	DGDG	Limited	
37	DGDG	Limited	
38	DGDG	None	
39	MGDG	Exchange	Leaves the PLQ exchange cavity and gets replaced by two MGDG lipids
40	MGDG	Limited	
41	MGDG	Exchange	Gets replaced by weak binding lipids including MGDG
42	MGDG	Exchange	Gets replaced by weak binding lipids including MGDG
43	DGDG	Exchange	Gets replaced by a strong binding MGDG lipid.
44	DGDG	Escape	Escape seems to be unrelated with movement of PsbX. No density visible in Figure 3.
53	MGDG	Limited	
54	MGDG	Limited	A second MGDG enters the PLQ exchange cavity
57	DGDG	None	
58	DGDG	None	

[a] *Numbering as in Fig. 3. The lipid mobility has been classified as 'none' or 'limited' for lipids that show no or very limited movement, 'large' for lipids that show a significant displacement and 'escape' for lipids that leave the binding site. In case an escaped lipid is replaced by another lipid, it is classified as 'exchange'. Note that nr. 15 and 16 are not included in this list, because they are beta carotenes.*

Table S3: Overview of the number of co-crystallized lipids per mobility category.[a]

Lipid	Total Number	None	Limited	Large	Exchange	Escape
				Total		
PG	12	4 (33%)	7 (58%)	1 (8%)	-	-
DGDG	24	12 (50%)	2 (8%)	-	4 (17%)	6 (25%)
MGDG	12	2 (17%)	5 (42%)	-	3 (25%)	2 (17%)
SQDG	12	2 (17%)	7 (58%)	2 (17%)	-	1 (8%)
				Stromal		
PG	12	4 (33%)	7 (58%)	1 (8%)	-	-
DGDG	2	2 (100%)	-	-	-	-
MGDG	2	-	-	-	-	2 (100%)
SQDG	12	2 (17%)	7 (58%)	2 (17%)	-	1 (8%)
				Lumenal		
PG	-	-	-	-	-	-
DGDG	22	10 (45%)	2 (9%)	-	4 (18%)	6 (27%)
MGDG	10	2 (20%)	5 (50%)	-	3 (30%)	-
SQDG	-	-	-	-	-	-

[a]*Numbers are shown for each leaflet as well as totals for the complete bilayer. For each lipid, the percentages for the mobility types are shown between brackets.*

Table S4: Lipid diffusion of co-crystalized and membrane lipids in and out of the PLQ exchange cavity.[a]

Monomer	PG		DGDG		MGDG		SQDG	
	in	out	in	out	in	out	in	out
				Stromal				
left	-	-	-	-	1 (I)	1 (I)	1 (I)	-
right	-	-	-	-	1 (III)	1 (III)	-	-
				Lumenal				
left	-	-	-	-	1 (I)	-	-	-
right	-	-	-	1 (III)	4 (III)	3 (III)	1 (III)	1 (III)

[a] *The number of lipids that enter and leave the PLQ exchange cavity is discriminated for each leaflet and monomer. The channel used by the lipids is shown between parentheses. Channel I refers to the PLQ channel as described in (2). Channel III is located between PsbJ on one side and between PsbK and ycf12 on the other side, and opens when PsbJ moves towards Cyt b 559α (3). 'Left' refers to the monomer in Figure 3 where the bindings sites are marked with an '*' and 'right' to the monomer where the binding sites are marked with a '#'.*

Supplementary Figures S1-S2

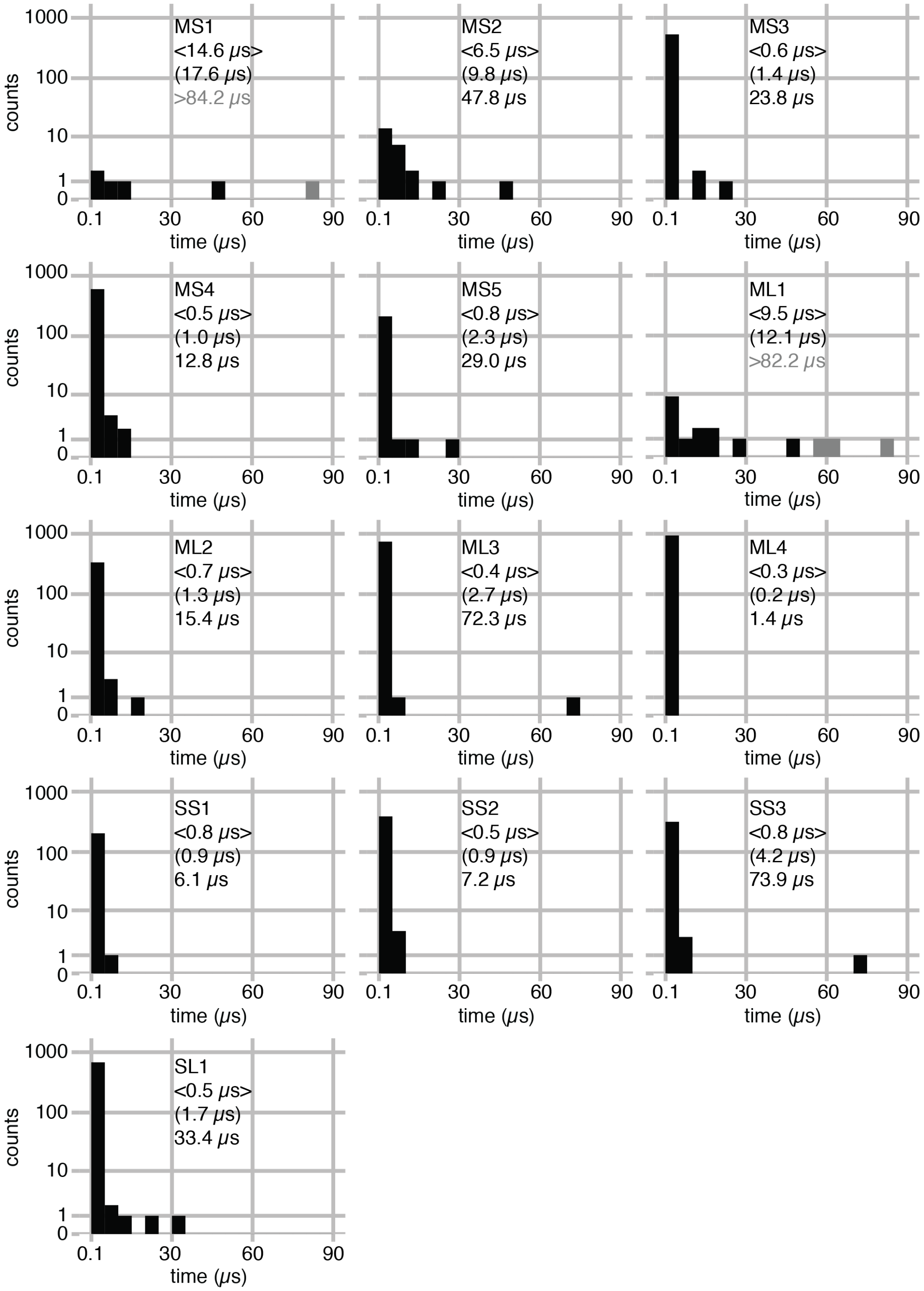

Figure S1: Lipid residence times in the different lipid binding sites. *For each binding site there is a histogram, with on the x-axis the residence time and on the y-axis the number of binding events (note that a single lipid could bind multiple times). The inset in each histogram shows the name of the binding site, the average residence*

time, its standard deviation and the longest residence time for that site. Residence times of lipids that are still bound at the end of the simulation and that were bound for at least 50 μs are coloured grey and not included in the calculation of the average residence time. If this lipid has the highest residence time of its site, the longest residence time in the inset represents a lower limit and is coloured grey as well. Note that a lower cut-off of a residence time of 100 ns was applied. The graphs and averages are calculated using the combined data of the two monomers.

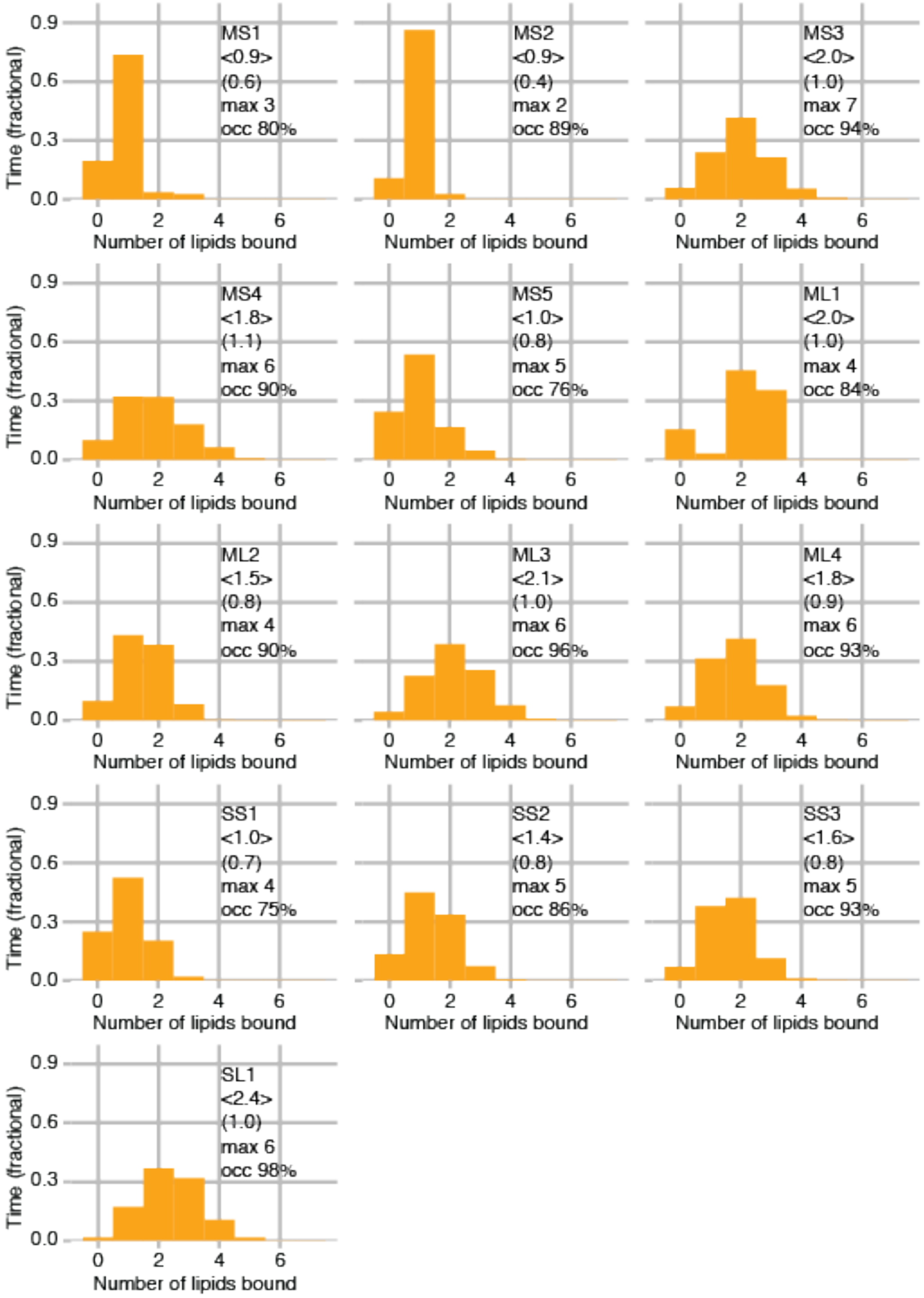

Figure S2: Lipid occupancy per binding site. *For each binding site there is a histogram, with on the x-axis the number of lipids bound and on the y-axis the fractional simulation time. The inset in each histogram shows the name of the binding site, the average number of lipids bound, its standard deviation, the maximum number of lipids bound for that site and the occupancy percentage. The data for each binding site represents the average over the two monomers, except for the maximum numbers of lipids bound, which is the maximum over the two monomers. Note that all lipids that bind were included, i.e., no lower cut-off of a 100 ns residence time was used.*

Supplemental References

1. Guskov, A., J. Kern, A. Gabdulkhakov, M. Broser, A. Zouni, and W. Saenger. 2009. Cyanobacterial Photosystem II at 2.9-Å Resolution and the Role of Quinones, Lipids, Channels and Chloride. Nat. Struct. Mol. Biol. 16: 334–342.

2. Loll, B., J. Kern, W. Saenger, A. Zouni, and J. Biesiadka. 2005. Towards complete cofactor arrangement in the 3.0 Å resolution structure of photosystem II. Nature. 438: 1040–1044.

3. van Eerden, F.J., M.N. Melo, P.W.J.M. Frederix, X. Periole, and S.J. Marrink. 2017. Exchange pathways of plastoquinone and plastoquinol in the photosystem II complex. Nat. Commun. 8: 15214.
