# Supplementary material for: Prediction of Thylakoid Lipid Binding Sites on Photosystem II
Source: Biophys J. 2017 Dec 19;113(12):2669–81. doi: 10.1016/j.bpj.2017.09.039 (PMC5770566; doi:10.1016/j.bpj.2017.09.039)
Supplement: Document S2. Article plus Supporting Material [file mmc2.pdf]

Biophysical Journal
Article

Biophysical Society

Prediction of Thylakoid Lipid Binding Sites on Photosystem II

Floris J. Van Eerden,[1,*] Manuel N. Melo,[1,2] Pim W. J. M. Frederix,[1] and Siewert J. Marrink[1]
[1]Groningen Biomolecular Sciences and Biotechnology Institute and Zernike Institute for Advanced Materials, University of Groningen, Groningen, the Netherlands and [2]Instituto de Tecnologia Química e Biológica António Xavier, Universidade Nova de Lisboa, Oeiras, Portugal

ABSTRACT The thylakoid membrane has a unique lipid composition, consisting mostly of galactolipids. These thylakoid lipids have important roles in photosynthesis. Here, we investigate to what extent these lipids bind specifically to the Photosystem II complex. To this end, we performed coarse-grain MD simulations of the Photosystem II complex embedded in a thylakoid membrane with realistic composition. Based on >85 μs simulation time, we find that monogalactosyldiacylglycerol and sulfoquinovosyldiacylglycerol lipids are enriched in the annular shell around the protein, and form distinct binding sites. From the analysis of residue contacts, we conclude that electrostatic interactions play an important role in stabilizing these binding sites. Furthermore, we find that chlorophyll *a* has a prevalent role in the coordination of the lipids. In addition, we observe lipids to diffuse in and out of the plastoquinone exchange cavities, allowing exchange of cocrystallized lipids with the bulk membrane and suggesting a more open nature of the plastoquinone exchange cavity. Together, our data provide a wealth of information on protein-lipid interactions for a key protein in photosynthesis.

INTRODUCTION

PSII is a major player of the photosynthetic machinery, the process by which light is converted into chemical energy in plants, algae, and cyanobacteria. PSII functions as a homodimer in vivo; each monomer consists of 27 subunits in plants and 20 in cyanobacteria, respectively. A large number of cofactors supplement PSII with its light harvesting and water splitting capabilities (1,2).

PSII is located in the thylakoid membrane, together with photosystem I (PSI), cytochrome b_6f complex, and ATP synthase, the other major proteins involved in photosynthesis. Over the last few decades, it has become apparent that there is a complex interplay between lipids and proteins. Lipids are vital for the functioning of proteins, and they have structural as well as functional roles (3). The thylakoid membrane is atypical as it has a rather unusual composition, containing mainly galactolipids instead of the more common phospholipids. The predominant galactolipids found in the thylakoid membranes of higher plants and cyanobacteria are digalactosyldiacylglycerol (DGDG), monogalactosyldiacylglycerol (MGDG), and sulfoquinovosyldiacylglycerol (SQDG), together with phosphatidylglycerol (PG) (4,5). Although the role of these lipids is poorly understood, it is clear that they are essential for the photosynthetic process. First of all, the composition of the thylakoid membrane is conserved in evolution (5), indicating the importance of the galactolipids for photosynthesis. Secondly, experiments show that the lipid composition influences the dimerization state of the protein and also the attachment of the inner antenna proteins, which are important for the electron transport within PSII (6).

MGDG is the most abundant lipid in the thylakoid membrane and is present in the crystal structures of both PSI and PSII (7–9). It is a non-bilayer-forming lipid that prefers the inverted hexagonal phase when isolated (10,11), which might be vital for the structural flexibility of the thylakoid membrane (12) and important for the functioning of the violaxanthin cycle (13,14). MGDG is found at the dimer interface of PSII and it promotes the dimerization of PSII (6,9). MGDG has additional roles in the functioning of the antenna complexes (15,16), and is important for chloroplast development (17).

DGDG is also a major component of the thylakoid membrane and is found in the crystal structure of PSII, which contains five DGDG molecules (8). Like MGDG, DGDG has a major influence on structure and functioning of the antenna complexes (16,18,19). Absence of DGDG leads to the detachment of the external subunits from PSII, resulting in a decreased oxygen-evolving capacity of the photosystem (20). The existence of hydrogen bonds between DGDG

Submitted June 28, 2017, and accepted for publication September 29, 2017.
*Correspondence: floris@vaneerden.eu
Editor: Markus Deserno.
https://doi.org/10.1016/j.bpj.2017.09.039
© 2017 Biophysical Society.
This is an open access article under the CC BY license (http://creativecommons.org/licenses/by/4.0/).
CrossMark

and PSII tyrosines has been shown (21). DGDG has been further shown to influence the thylakoid membrane ultrastructure (22–25) and lipid packing, the latter playing a role in heat resistance (26).

Although PG is only a minority component of the thylakoid membrane, it has a number of important roles. PG lipids sustain the electron transport activity of PSII, possibly through induced conformational changes at the Q_B site (6,27–31). PG has also been implicated in PSII dimerization (32,33) via stabilization of the attachment of the external subunits PsbO, PsbU, and PsbV, which are necessary for dimerization of PSII; a lack of PG could therefore indirectly lead to the monomerization of the PSII complex (34,35). PG is furthermore involved in the stability of PSI and the antenna supercomplexes (16,18,19,36,37). During phosphate starvation, PG can be replaced by DGDG (38) or SQDG (39).

The anionic SQDG is also relevant for photosynthesis, with four molecules being found in the crystal structure of PSII from cyanobacteria (8). Absence of SQDG results in impaired electron flow from water to tyrosine Z, possibly due to conformational changes in PSII caused by the absence of SQDG (40–42). SQDG furthermore influences the assembly of the PSII subunits, including the antenna complexes (6,16).

In addition to the headgroups, the lipid tail composition of the thylakoid membrane is highly regulated, in particular the number and position of double bonds. The saturation grade of the thylakoid membrane differs per organism (5). The presence of unsaturated lipid tails is especially important for photosynthesis (40,43,44). Simulation studies (45) show that the unsaturated fatty acids keep the thylakoid membrane of plants in a fluid phase at physiological temperatures. For cyanobacteria, which function at elevated temperatures, this is not required.

Taken together, the experimental data show that thylakoid lipids can exert their effect at different levels of the photosynthetic process. This includes influencing the dimerization of PSII monomers, stabilizing the interactions between the various subunits, facilitating the electron transport, and maintaining the structural integrity of the complex toward the reorganization of antenna complexes. However, due to the difficulty to directly probe lipid-protein interactions, it remains unclear how the thylakoid lipids affect the functioning of PSII (6). Computer simulations are a useful tool to complement experiments in this regard (46–48). Previous MD simulations have revealed preferential lipid-protein interactions and lipid binding sites on a large variety of membrane proteins (49–56). PSII has also been subjected to computational modeling (57–61), but the interaction with the thylakoid lipids has not been investigated so far.

Here we fill this gap based on coarse-grain (CG) MD simulations of PSII embedded in a realistic model of the thylakoid membrane. By omitting atomistic detail, CG models allow us to probe the behavior of a system on much longer timescales (62). Our model is based on the cyanobacterial PSII structure, which was resolved by x-ray crystallography by Umena et al. (8). The PSII cores of plant and cyanobacteria are highly similar; any large differences are mainly found in the lumenal subunits and in the antenna systems (63). The thylakoid lipid composition also differs slightly in plants and cyanobacteria (5,45). We previously reported on the overall dynamics of the cyanobacterial PSII complex including all of its cofactors (64) and on the exchange of the electron carriers plastoquinone and plastoquinol (65). Here we focus on the interaction of the PSII complex with the thylakoid lipids. We find an overall enrichment of MGDG and SQDG lipids around the complex, and identify a number of potential lipid binding sites on the membrane-exposed surface of PSII. We discuss the nature of these binding sites and their biological relevance in relation to the available experimental data.

METHODS

System setup

The MD trajectory analyzed in this article is the same as described previously (64). (For a more detailed description of the system setup, we refer the reader to the Methods of van Eerden et al. (64).) In short, the simulations were based on the PSII x-ray structure of the thermophilic cyanobacterium *Thermosynechococcus vulcanus*, PDB: 3ARC (8). All cocrystallized lipids were included in the system, as well as all cofactors. This structure was embedded in a realistic representation of the thylakoid membrane consisting of 2686 lipids using the Insane script (66); see Fig. 1. The membrane is composed of 18:1 (9)–16:0 MGDG (40 mol %), 18:1 (9)–16:0 DGDG (25 mol %), 18:1 (9)–16:0 SQDG (15 mol %), di16:0 SQDG (10 mol %), and 18:1 (9)–16:0 PG (10 mol %) lipids, following the experimental data on thylakoid membrane composition of cyanobacteria (5). The system was solvated with 73,144 CG water molecules (representing four times as many water molecules), then 455 Na^+ and 455 Cl^- ions were added, corresponding to $\sim$100 mM Na^+Cl^-, plus 1032 Na^+ counterions to neutralize the overall charge of the protein and the negative charge on the PG and SQDG headgroups.

The interactions were modeled with the Martini force field (67), using version 2.2 for the protein complex (68) in conjunction with the ElNeDyn elastic network to stabilize the tertiary structure of the protein subunits (69). The lipid parameters for the galactolipids were taken from (70) with the modifications as described in (45). The GROMACS 4.5.5 MD package (http://www.gromacs.org/) was used to perform the MD simulations with the common settings defined for the Martini force field (71). The system was simulated in the isothermal-isobaric (NpT) ensemble. The temperature was set to 328 K using the V-rescale thermostat with a coupling constant $\tau_t = 2.0$ ps (72). The pressure was coupled semiisotropically to an external bath of $p = 1$ bar with a coupling constant of $\tau_p = 1.0$ ps and a compressibility of $\chi = 3.0^{-4}$ bar^{-1} using the Berendsen barostat (73). Electrostatic interactions were calculated using a shifted potential with a cutoff of 1.2 nm in conjunction with a dielectric constant of 15. The van der Waals interactions were also calculated using a shifted potential, with a cutoff of 1.2 nm and a switch at 0.9 nm. The original simulation of 60 μs (64) was extended to 88.7 μs. The first microsecond was discarded as equilibration, yielding a total simulation time used for analysis of 87.7 μs.

Lipid density maps and definition of binding sites

To identify preferential enrichment of certain lipids around the protein, the average lateral density distributions of the lipids were calculated—the

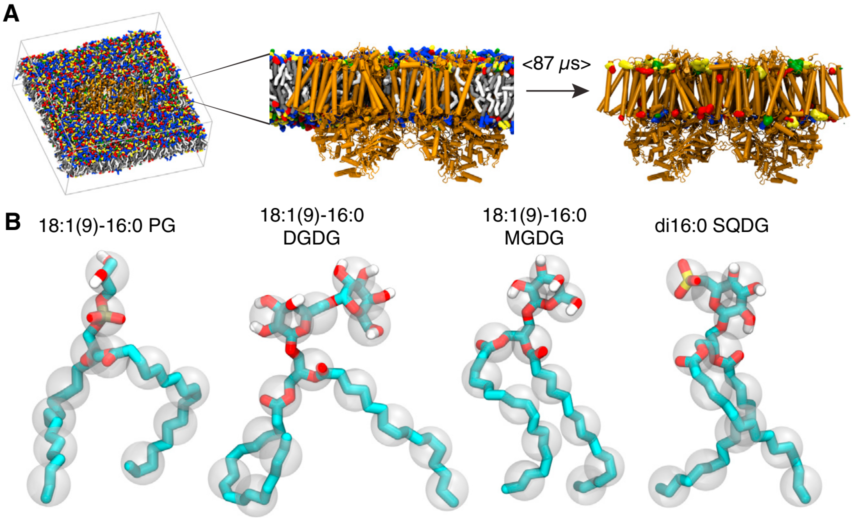

FIGURE 1 System setup. (*A*) Given here is an overview of the simulation protocol. The left panel shows a snapshot of the simulation box of PSII embedded in the thylakoid membrane, with cofactors and solvent omitted for clarity. Lipid headgroups are colored as follows: PG, green; DGDG, blue; MGDG, red; SQDG, yellow. The center panel shows a side view of the equilibrated PSII dimer. When the lipid positions are averaged over the whole simulation (87 μs), lipid occupancies can be calculated and regions of high lipid occupancy become visible as lipid binding sites, as shown in the right panel. (*B*) Shown here are atomistic structures of the thylakoid lipids with the coarse-grained beads superimposed; aliphatic hydrogens are omitted for clarity. To see this figure in color, go online.

so-called "density maps". The relative densities of the thylakoid lipids around the protein were calculated using the g_mydensity tool (74). To prevent artifacts from the periodic boundary conditions, jumps were removed from the simulation and the simulation box was quadrupled. The quadrupled trajectory was then centered on one of the four PSII subunits, and subsequently translationally and rotationally fitted on the centered PSII. The positions of the GL1 glycerol beads were used to represent the lipids. Both membrane lipids and cocrystallized lipids were included in the analysis. For each leaflet, the densities were calculated separately. The densities were normalized according to the average density of the lipid species in the entire membrane plane.

Potential binding sites were identified by calculating the time-averaged occupancies of headgroups throughout the simulation box. This was done using the Volmap tool of the VMD visualization package, which overlays a grid on the system and counts the occupation of each cell. The grid resolution was set to 0.2 nm and counting was performed after fitting the simulation on the protein backbone beads (75). The threshold for showing occupancies was based on the amount of MGDG binding sites and adjusted for the other lipids. The MGDG densities reveal clear spots in the simulation box with a threshold value of 36% (implying that at least in 36% of the frames a MGDG headgroup is present, which corresponds to roughly seven times the occupancy of MGDG in the bulk). We adjusted this value for the other lipids based on their relative abundance in the membrane and their headgroup size, which resulted in the following thresholds: 6% for PG, 45% for DGDG, and 30% for SQDG headgroups. Please note that not all grid points in the simulation box are occupied by a lipid in every single frame. Therefore, the bulk occupancy is not the same as the bulk concentration. Only binding sites that were observed in both monomers were selected. The last snapshot of Fig. 1 illustrates the iso-occupancy surfaces thus obtained.

Composition of binding sites

The amino acid composition of the binding sites was determined according to the procedure suggested by Arnarez et al. (55). At 10 ns intervals, for every lipid we created a so-called "phrase", which is a list of protein residues found within 0.8 nm of the lipid headgroup at that time point. We only considered phrases that contain at least five residues. Subsequently the phrases were clustered with a GROMOS-like algorithm (55,76). At the start of the clustering procedure all phrases are pooled. Phrases of which 70% of the residues are in common are clustered and the biggest cluster is removed from the pool. This is repeated until all phrases are clustered. Residues that are present in at least 80% of the phrases in a cluster are selected as part of the binding site. All binding sites were inspected manually. If it appeared that residues did not actually contribute to the site, but were merely a "by-catch" of the algorithm, they were removed from the binding site. Two examples are residues in the periphery of a binding site that only contribute marginally to the binding of lipids or residues that are part of a flexible loop, which occasionally diffuses toward the binding site, and thereby only temporarily becomes part of the binding site. An estimate of the uncertainty of the composition was calculated by considering three blocks of ~29 μs as quasi-independent samples as well as each monomer, resulting in a sample size of six. Please note that this is not a robust error estimate; due to the long correlation times present in the system (exceeding the total simulation time), independent block averages could not be obtained. Consequently, we note that the composition of many binding sites is not completely identical over the two monomers. For the final binding site composition, we combined the compositions of the binding sites of the two monomers to improve our statistics, e.g., if the composition of site X in the one monomer was Arg, Lys, and Tyr, and in the other monomer Lys, Tyr, and Asn, the final composition became Arg, Lys, Tyr, and Asn.

We normalized the amino acid composition of the binding sites by dividing the result of the procedure above by the composition of the residues that come into contact with any lipid headgroup during the simulation. An estimate for the latter was obtained by counting all residues that are at some point of the simulation within 0.8 nm of any (PG, DGDG, MGDG, or SQDG) lipid headgroup. In the normalized distribution, an occurrence >1 corresponds to a preference for a certain residue in a binding site, whereas an occurrence <1 would mean the residue is avoided in the binding site.

Lipid residence times

To calculate the lipid residence times, we monitored, with a frequency of once per nanosecond, whether a lipid was in contact with a residue from a binding site. We used a double cutoff method in combination with a threshold. The double cutoff method is used to prevent the so-called "rattling in a cage" motion that would yield artificially high counts of short residence periods; we used an inner cutoff of 0.6 nm and an outer cutoff of 1.6 nm. A lipid was considered to be in contact with the binding site once it was within the inner cutoff of any binding site bead, until it moved further away than the outer cutoff of any bead in the binding site. We also used a threshold that the lipid headgroup has to be in contact with at least 20% of all the binding site beads to remove lipids that only bind peripherally to the binding site. To filter out membrane lipids that just bypass the binding site without actually binding, we only included residence times of at least 100 ns in the calculation of the average residence time and its standard deviation. Lipids that had been bound for >50 μs and were still

bound at the end of the simulation were also excluded from the average value, because their residence time represents only a lower limit.

RESULTS

We simulated the PSII dimer embedded in the thylakoid membrane over a time period of 87.7 μs. This timescale is long compared to the diffusion rate of individual lipids in the thylakoid membrane; a displacement of 1 nm takes, on average, ~5 ns (45). The long timescale allowed us to analyze the preferential binding of thylakoid lipid types to the PSII complex. We first present results on the specific enrichment and depletion of these lipids based on density maps. We continue to identify specific lipid binding sites based on density iso-surfaces, and characterize the binding sites in terms of residue content and lipid residence times. Finally, a qualitative description is made of the behavior of the cocrystallized lipids.

Please note that we will use the stromal and lumenal nomenclature to refer to the inside and the outside, respectively, of the thylakoid membrane. Strictly speaking, this is only valid for plants, as the cyanobacterial thylakoid membranes are directly located in the cytoplasm and not, as in plants, inside the chloroplast.

MGDG and SQDG enriched around PSII

To probe the preference of PSII for specific lipids, we computed lipid density maps (see Methods). The density maps for the four classes of lipids (MGDG, DGDG, PG, and SQDG) are shown in Fig. 2. Remarkably, the density maps are quite different for each lipid. MGDG and SQDG are clearly enriched around the protein, showing several regions of high densities in the annular lipid shell. This is true for both the stromal and lumenal leaflets. PG and DGDG, on the other hand, are depleted from this region, with the exception of some high-density spots inside the protein that originate from cocrystallized lipids. In particular, PG appears to be excluded from the annular lipid shell, but also to have a somewhat increased density in the subsequent lipid layers. The densities of the cocrystallized lipids in the plastoquinone exchange cavities are also visible. Interestingly, for all lipids but PG the densities inside the cavities are connected with those in the membrane (see the *arrows* in Fig. 2), implying that lipids are able to exchange between the plastoquinone exchange cavity and bulk membrane.

MGDG and SQDG occupy distinct binding sites at membrane-exposed surface

To identify possible binding sites, we calculated iso-occupancy surfaces of the lipid headgroups around the PSII dimer complex. Using a threshold occupancy of five times the bulk density, we obtained clear spots of preferred interaction of lipids with the protein (see Methods). Spots that were present symmetrically, i.e., observed in both monomers, were classified as lipid binding sites. Using this procedure, we found 13 distinct binding sites at the membrane-exposed surface of PSII, in addition to the sites reflecting the cocrystallized lipids. All sites are indicated in Fig. 3 and listed in the Supporting Material (Tables S1 and S2).

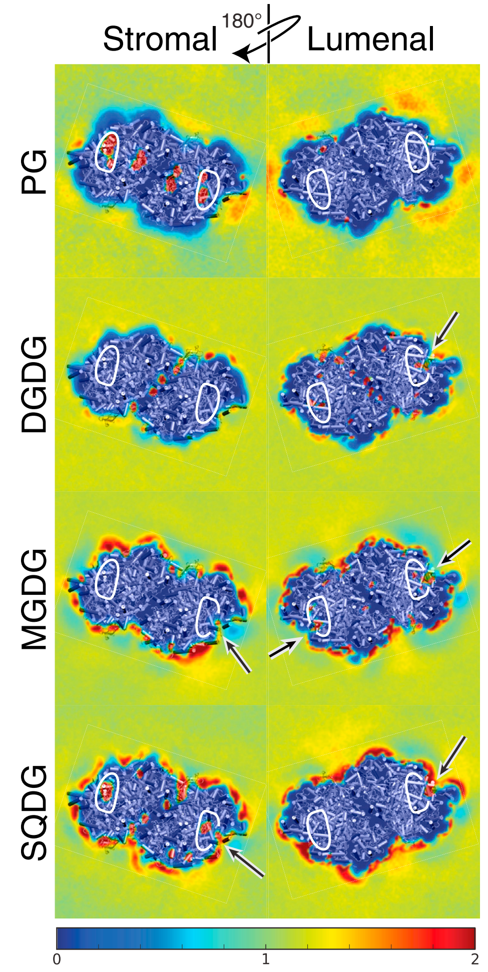

FIGURE 2 Normalized density maps of the four different thylakoid lipids. A density of 1 corresponds to the average (bulk) density in the membrane plane. Densities have been capped at two times the bulk density. A cartoon representation of PSII is included to indicate its approximate position and orientation. The location of the plastoquinone exchange cavity is indicated by white lines. Arrows indicate connection points between the high lipid density regions in the plastoquinone exchange cavity and in the membrane; for clarity, the white lines are omitted here. To see this figure in color, go online.

In line with the enrichment of MGDG and SQDG, and depletion of DGDG and PG around the protein (Fig. 2), peripheral binding sites are only found for MGDG and SQDG, with nine and four sites, respectively. There are a few spots where PG and DGDG bind, but they are never present in both monomers and therefore do not match our definition of a binding site. For SQDG, three out of four binding sites are located at the stromal side, as are all the SQDG cocrystallized lipids. MGDG has binding sites on both sides of the

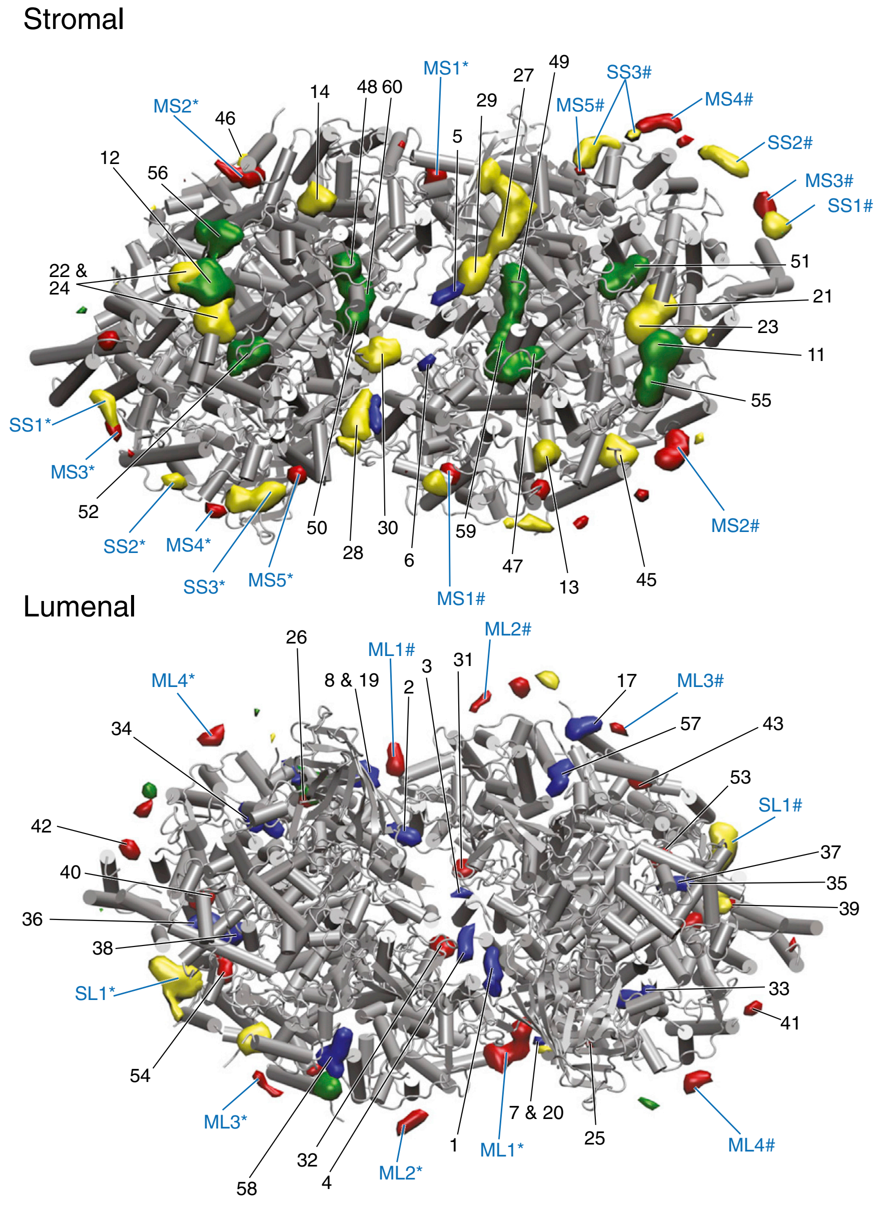

FIGURE 3 Identification of lipid binding sites. Given here are stromal and lumenal views of the PSII backbone with lipid binding sites indicated. PG binding sites are depicted in green, DGDG in blue, MGDG in red, and SQDG in yellow. Binding sites resulting from cocrystallized lipids are numbered in black and refer to Table S2. Binding sites originating from membrane lipids, and which are present in both monomers, are labeled in blue. Labeling of the sites is given according to lipid type (first letter: M, MGDG; S, SQDG), membrane side (second letter: *L*, lumenal; *S*, stromal), and sequential numbering. To distinguish between the mirror sites in the two monomers, binding sites in the left monomer are marked with an "*" and in the right monomer with a "#". To see this figure in color, go online.

protein (five stromal versus four lumenal), although the majority of its cocrystallized lipids are located on the lumenal side.

The binding sites appear fairly homogeneously distributed around the entire membrane-exposed surface of the protein. However, with the exception of a SQDG lumenal binding site (SL1), there are no binding sites around subunits Cyt b 559α, PsbJ, ycf12, and PsbZ. The slightly higher mobility of these subunits (64) might prevent the formation of a well-defined binding site in this region.

Charged residues important for binding

To characterize the nature of the membrane-exposed binding sites, we analyzed the residues comprising those sites (see Methods). The full list of residues for each site is shown in Table S1. The statistical distribution of these residue types is shown in Fig. 4, as well as the normalized distribution corrected for the prevalence of the residues in the protein.

Charged residues appear to be important components of the binding sites; in fact, 12 out of 13 binding sites contain at least one charged residue. Consistently, the binding sites of SQDG ($-1e$ charge) only contain cationic residues (Arg, Lys), whereas sites of MGDG (neutral) contain both negative and positively charged amino acids (Arg, Lys, Asp, Glu). When considering the normalized amino acid composition, in the MGDG binding sites the two positively charged amino acids (Arg and Lys) are equally present. However, this is not true for the negatively charged residues, where aspartic acid is more abundant than glutamic acid in MGDG sites. The importance of electrostatic interactions is

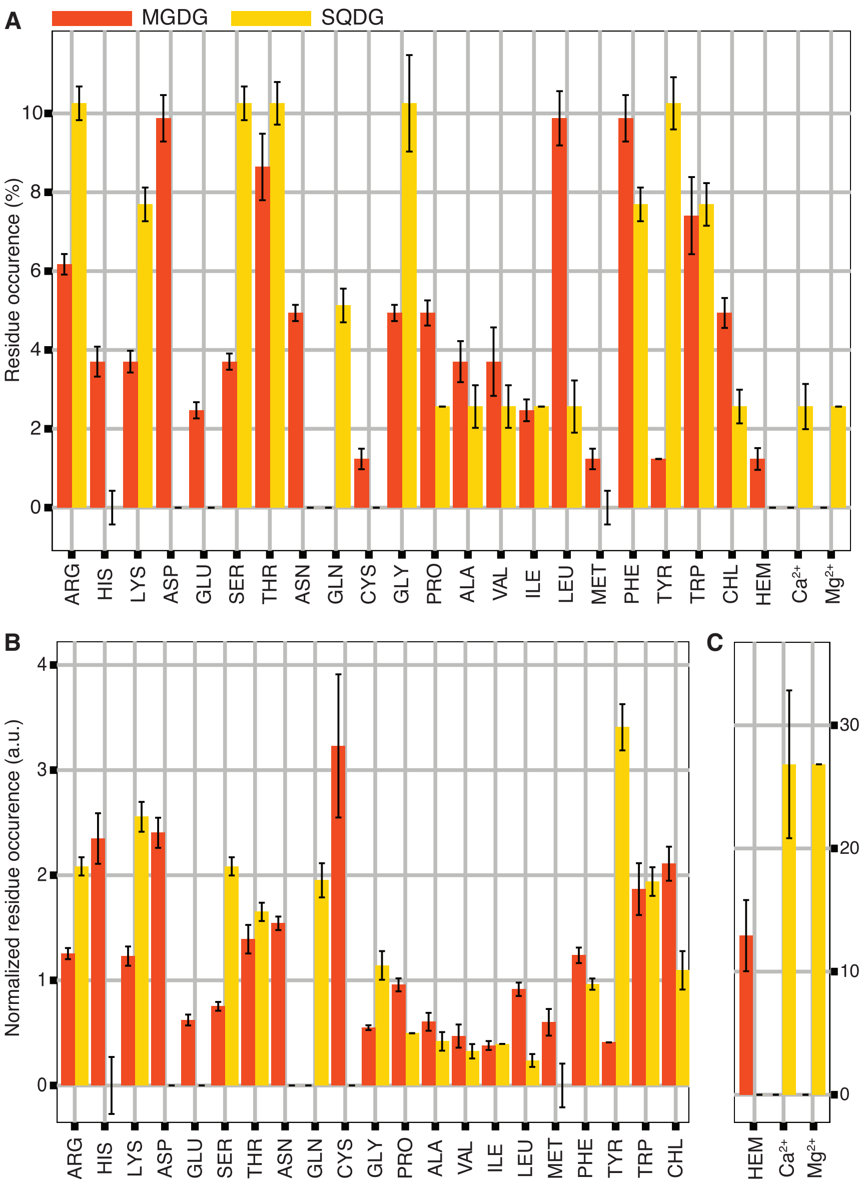

FIGURE 4 Binding site composition. Given here are bar plots with the amino acid and cofactor composition of the MGDG (*red*) and SQDG (*yellow*) binding sites. (*A*) Shown here is the relative occurrence in terms of percentage. In total, there are 81 residues comprising the MGDG sites and 39 comprising the SQDG sites. This means that an occurrence of respectively 1.2% for MGDG and 2.6% for SQDG sites corresponds to the presence of only one amino acid of that type across all the binding sites. (*B* and *C*) Given here is the normalized occurrence of amino acids and cofactors. The occurrence of heme, Ca^{2+}, and Mg^{2+} is shown in a separate plot with a different scale to improve readability. Error bars represent an estimate of the uncertainty (see Methods). To see this figure in color, go online.

further underlined by the occurrence of the cofactors chlorophyll *a* (CHL), heme, and cocrystallized ions in the binding sites. CHL does contain charged beads, but has a net charge of zero. This might explain why CHL is prevalent in the sites of the neutral MGDG rather than in those of the negatively charged SQDG lipid. Apart from this, some of the binding sites containing CHL, e.g., MS1 and ML1, are located toward the center of the bilayer preventing binding of charged lipids such as SQDG. Heme carries a net charge of $-2e$, and is not found in the SQDG binding sites. The SL1 sites, however, do contain a Ca^{2+} and a Mg^{2+} ion, which form a salt bridge with the SQDG lipids.

The polar threonine and the aromatic phenylalanine residues are enriched in both MGDG and SQDG binding sites, as well as tryptophan, serine, and glycine. Threonine and serine are both polar amino acids with clear hydrogen bonding capacities, and therefore interact favorably with the many hydroxyl groups of the MGDG and SQDG lipids. Although asparagine and glutamine are polar as well, asparagine only occurs in MGDG and glutamine in SQDG sites. Aromatic amino acids prevail at the interface between a polar membrane and aqueous solvent, which is also the location of the binding sites. One could therefore argue that aromatic residues are enriched in the binding sites, just because they share the same location in the protein. However, in the normalized distributions, the aromatic residues stand out, especially tryptophan, and in the SQDG sites, tyrosine as well. It is unlikely that the hydrophobic effect is solely responsible for the enrichment of aromatic residues in binding sites, because there are many other hydrophobic residues present at the membrane interface. It remains therefore unclear what drives the favorable

interaction between these aromatic residues and the thylakoid lipids.

Residues like leucine and glycine are, in absolute number, a big contributor to the binding sites. After normalizing for their occurrence in the protein, the contribution of leucine and glycine to the binding sites seems less prominent and more based on their ubiquity in the membrane-exposed surface of the protein than due to specific interactions with the thylakoid lipids.

Lipid residence times vary by orders of magnitude

To characterize the kinetics of the lipids in the binding sites, we analyzed the lipid residence times. To quantify the residence times, we counted how long lipids stay within 1.6 nm of the binding site from the moment the lipids entered within a 0.6-nm cutoff radius of the binding site (see Methods). The distribution of residence times of the MGDG and SQDG lipids is shown in Fig. 5 *A*. A breakup per individual binding site can be found in Fig. S1.

We observed a broad range of residence times, varying over at least three orders of magnitude from 100 ns to at least 86 μs (i.e., limited only by the length of our trajectory). The vast majority of MGDG and SQDG lipids have residence times below 1 μs. Using a threshold residence time of 100 ns to filter out transiently passing membrane lipids, we calculate an average residence time of 0.5 μs for both MGDG and SQDG, with SDs of 2.5 and 1.9 μs, respectively. There are only a few lipids that remained in their binding pocket longer than 30 μs, and most of them are MGDG lipids. One MGDG lipid stayed bound during the entire trajectory, implying a residence time >86 μs. For SQDG, the longest binding time observed is 74 μs. Considering the total bound time of the lipids (obtained by multiplying the number of bound lipids in a given residence time interval with the residence time; Fig. 5 *B*), it is clear that a significant fraction of the total bound lipid time can be attributed to lipids with long residence times.

Most of the binding sites bind multiple lipids for a relatively short time, e.g., MS3, MS4, MS5, ML2, ML3, ML4, SS1, and SS2. There are, however, two binding sites that bind a single or a few lipids for an elongated time, sites MS1 and ML1, depicted in Fig. 6. These two sites are both located at the dimer interface. In both of these sites, CHLs play an important role in the binding of the lipids. Whereas MS1 mostly coordinates a single lipid, ML1 is occupied by two or three lipids. The sites MS2, SS3, and SL1 form an intermediate category that bind lipids for a significant amount of time, but at the same time bind many lipids for a short time. SL1 is of particular interest, because it is located at plastoquinone (PLQ)/plastoquinol (PLQol) exchange channels I and III (9,65) and might therefore have an influence of the PLQ/PLQol exchange (Fig. 6). It is

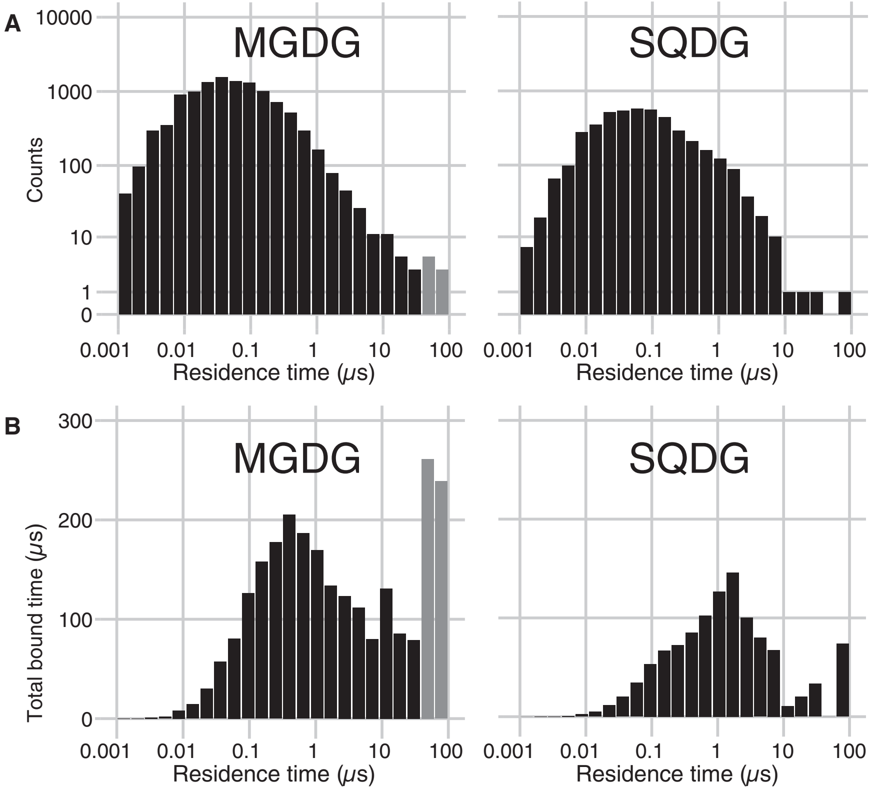

FIGURE 5 Distribution of lipid residence times. Logarithmically binned histograms of lipid residence times are shown for the MGDG (*left*) and SQDG (*right*) binding sites. Gray bars indicate bins containing lipids that were still bound at the end of our simulation and had been bound for at least 50 μs. In (*A*), the total count of binding events of a certain duration are shown. In (*B*), the value of each bar represents the summed time of the binding events in the respective bin (total bound time, i.e., the count multiplied with the residence time), giving proportional prominence to longer residence-time events, which are fewer in number but can represent most of the simulation time.

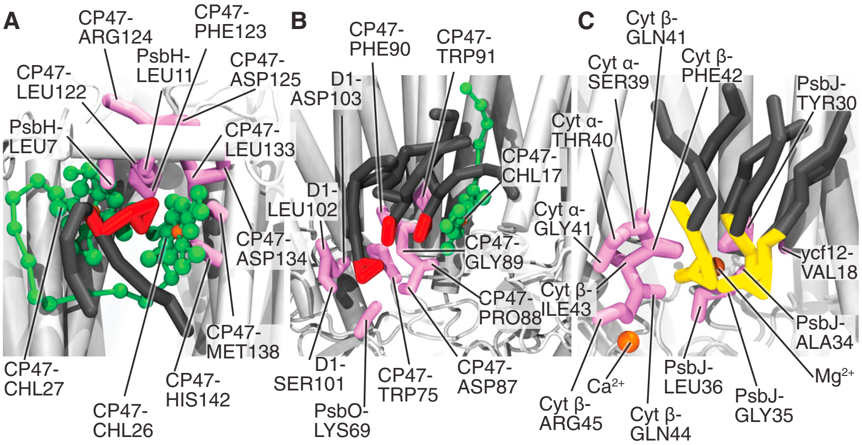

FIGURE 6 Visualization of key binding sites. Snapshots of binding sites MS1 (*A*), ML1 (*B*), and SL1 (*C*) are shown. The MS1 site consists of a MGDG lipid coordinated by chlorophylls (*green*) and various amino acids, the ML1 sites contain three MGDG lipids coordinated by amino acids and a single chlorophyll, and the SL1 site contains three SQDG lipids coordinated by amino acids and ions. MGDG headgroups are colored red and SQDG yellow; lipid tails are colored dark gray. The lipids are shown as sticks and the proteins are shown in white cartoon representation, with the binding-site amino acids as pink sticks, the CHLs in ball-and-stick representation in green, and the ions as orange spheres. To see this figure in color, go online.

also the only binding site to contain ions. For a more detailed description of every binding site, we refer the reader to Fig. S2.

Dynamics of cocrystallized lipids

Our simulation data also allow us to look at the mobility of the lipids that are found in the crystal structure. Based on visual inspection, we determined for all the cocrystallized lipids whether they stay in their binding site or diffuse into the membrane. We distinguish between lipids that show no or very limited movement with respect to their position in the x-ray structure ("none", "limited"), and between lipids that show larger displacements ("large") or even escape from their binding site ("escape"). In some cases, escaped lipids get replaced by other lipids ("exchange"). The result of this qualitative assessment is shown in Table S2 for each of the 60 cocrystallized lipids. The average behavior is summarized in Table S3.

From the 60 cocrystallized lipids, we found 16 lipids to diffuse away from their initial binding pocket during the entire simulation. Especially MGDG and DGDG lipids were found leaving their binding site (~30%), which seems caused by the fact that many MGDG and DGDG cocrystallized binding sites are located at the protein periphery. In six cases, the escaped lipid gets replaced by a similar lipid. In the other 10 cases, the binding site is simply lost. The mobility of the lipids located at the surface of the protein is greatly dependent on how well they are shielded from the membrane. The diffusion of PsbX (64) results in the exposure of lipid sites 18 and 46 to the membrane, resulting in their escape. The mobility of the lipids in the protein interior and at the dimer interface is very limited, and none of these interfacial lipids leaves the dimer interface.

Of particular interest is the behavior of the cocrystallized lipids in the PLQ exchange cavity. Some of these lipids have very limited mobility (lipids 11, 36, 37, 38, 40, 55, 56), whereas others are very mobile (lipids 12, 21, 22, 23, 24); see Tables S2 and S3 and Fig. 3. A few cocrystallized lipids also unbind, and are capable of leaving the PLQ cavity (lipids 35 and 39). In fact, we find multiple lipids to diffuse in and out of the PLQ exchange cavity; see Table S4 and Fig. 7. This is in line with the continuous densities between the membrane and the PLQ exchange cavity shown in Fig. 2. There are two pathways that the lipids use to enter and leave the PLQ exchange cavity. The first pathway (channel I) is the PLQ channel formed by Cyt b 559α and PsbJ (77). The second pathway is a new channel that we recently described as a potential exchange channel for PLQ and PLQol (65). We denoted the new pathway "channel III", as a second PLQ channel was already described by Guskov et al. (9). Channel III emerges when PsbJ moves toward Cyt b 559α and PsbK and ycf12 move in the opposite direction, creating a tunnel between PsbJ on the one side and PsbK and ycf12 on the other side. The sizes of channels I and III can vary (65), and during this simulation, channel III is larger in size than channel I, allowing more lipids to diffuse in and out of the protein. In total (counting both cocrystallized lipids and membrane lipids), channel I is used four times whereas channel III is used 12 times (Table S4). Out of the in-total 16 exchanges, nine were lipid entrances into the PLQ exchange cavity, whereas the remaining seven were lipid exits out of the PLQ exchange cavity. This corresponds to a flux of, respectively, 51 and 40 entrance and exit events per monomer ms^{-1}. MGDG enters and leaves the protein most often, followed by SQDG and DGDG. Remarkably, PG is not seen entering or leaving the PLQ exchange cavity during the simulation at all.

The exchange of lipids results in a change in the number of lipids in the cavity, and alters the composition. At the start of the simulation, each cavity contains three PG, two DGDG, two MGDG, and two SQDG lipids, whereas during the last 20 μs the average composition in each cavity is 3.0 PG, 1.5 DGDG, 3.2 MGDG, and 2.5 SQDG lipids. There is thus an increase of 1.2 lipids in the exchange cavity per monomer during the simulation.

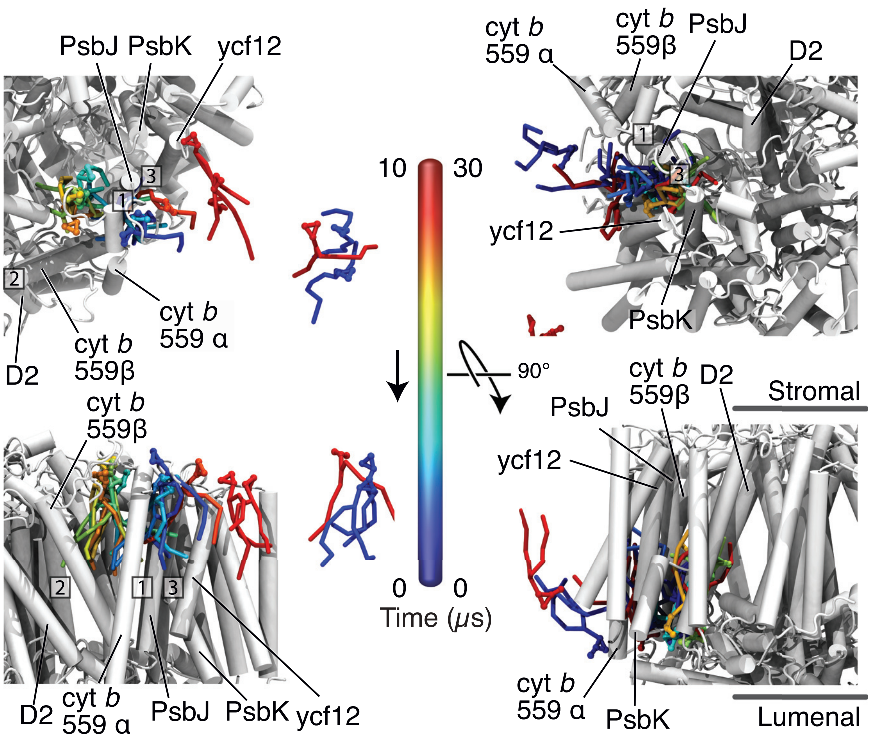

FIGURE 7 Lipid dynamics in the PLQ exchange cavity. Given here is a time series of snapshots of two diffusion events of a single MGDG lipid (*left*) and a SQDG lipid (*right*) into and out of the PLQ exchange cavity. The upper panels show a stromal view and the lower panels, a side view. The MGDG lipid originates from the stromal leaflet and diffuses through channel I, whereas the SQDG lipid originates from the lumenal leaflet and diffuses through channel III. The three PLQ channels are labeled, as well as the stromal and lumenal side of the protein. The color of the lipid is indicative of the relative time with respect to the first snapshot; the snapshots in the left and right panel are from a timespan of, respectively, ~10 and ~30 μs. In the upper-right panel, channel II is not visible and in the lower-right panel, all the three channels are not visible at the given viewpoints. To see this figure in color, go online.

DISCUSSION

Preferential interaction of PSII with MGDG and SQDG lipids

Lipids are of great importance for the functioning of membrane proteins, as has become clear in the ever-evolving picture of protein-lipid interactions (3). Our simulations provide a detailed view on how the complex PSII system interacts with the lipids constituting the thylakoid membrane and thereby help us to understand the functioning of this important protein. The major findings from the simulations are 1) the enrichment of MGDG and SQDG in the annular lipid shell around the protein, and 2) the occurrence of multiple MGDG and SQDG binding spots at the membrane-exposed surface. Some of these sites bind lipids for elongated times, in particular sites MS1, ML1, and SL1, which are occupied by at least one lipid most of the time (see Fig. 6). Other binding spots are in fact more transient binding regions that bind multiple lipids shortly, e.g., sites MS3, MS4, ML2, ML4, and SS1. Combining the peripheral cocrystallized sites, which are mostly stable in our simulations, with the new binding spots, we predict an average number of 31.0 MGDG (28.0 lipids from binding sites; see Fig. S2, plus 3.0 cocrystallized lipids, #41–43; see Table S2) and 13.8 SQDG lipids (12.8 lipids from binding sites, see Fig. S2; plus 1.0 cocrystallized, #45, see Table S2) bound to the membrane-exposed surface of the PSII dimer.

An intriguing question is, what are the driving forces leading to the binding site enrichment of MGDG and SQDG instead of DGDG and PG, two lipid types that are also present in large amounts in the thylakoid membrane? We attribute the relative depletion of DGDG to a combination of steric and entropic factors arising from its large and flexible headgroup, consisting of two sugar rings. MGDG, with only one sugar ring, can more easily approach the protein surface. In particular, chlorophyll has an important role in the coordination of MGDG lipids in some of the binding sites, e.g., MS1 and ML1. The outcompeting of PG by SQDG, when both carry a negative charge, is more puzzling. Positively charged residues are important components of the SQDG binding sites, but potentially PG lipids could also bind. A possible driving force is the general affinity of sugar residues for proteins, as is observed in, e.g., all-atom simulations of proteins in sugar solutions (78) and in CG simulations of proteins interacting with ganglioside lipids (79,80). Additionally, the lipid tails could play a role. SQDG is the only lipid in our simulated mixture that also occurs with fully saturated tails instead of one saturated and one unsaturated tail. However, further analysis of the SQDG binding sites does not reveal a strong preference for either the fully saturated or singly unsaturated variant (data not shown).

Putative biological role of binding sites

The observation of specific lipid binding sites warrants a discussion on their putative biological role. One possibility is that the lipid binding sites have a supporting or catalyzing function in the creation of PSII superstructures, by functioning as a bridge or glue between different complexes, including the associated antenna complexes as recently

shown in the formation of megacomplexes composed of PSI, PSII and a phycobilisome antenna complex (81). Additionally, CG MD simulations have shown the structural importance of lipids in supercomplex formation of respiratory chain complexes in mitochondrial membranes (55,82).

Stabilization of the dimer structure is another form of steering supercomplex formation. Of the two MGDG binding sites at the dimer interface, the ML1 site has contributions from residues originating from the two different monomers. The lipids in this site might therefore contribute to the dimerization. This is in agreement with experimental studies that have shown the importance of MGDG in PSII dimerization (6). The low mobility of the interfacial cocrystallized MGDG lipids 31 and 32 is also in agreement with the importance of interfacial lipids for dimerization. This might be also true for the low mobility of the interfacial DGDG lipids 1–6. Note that these lipids were detergents in the crystal structure that were modeled as DGDG molecules, but in vivo might actually be MGDGs.

Another possibility is that the lipids bound to the surface of PSII are important to sustain the electron transport in PSII, either by participating directly in the electron chain or by stabilization of other residues and molecules that take part in the electron transport. SQDG deficiency in *Chlamydomonas reinhardtii* results in a decreased efficiency of electron donation from water to Tyr Z (D1-TYR161) (42). It has been proposed that these changes in the electron donation could perhaps be the indirect result of changed interactions of the extrinsic proteins PsbO, PsbU, and PsbV (35). Although the residues of PsbV are not part of any binding site, during the simulation some PsbV residues interact with the SQDG lipids that reside at the SL1 site. Among the PsbV residues interacting with SQDG lipids are the charged residues PsbV-LYS24 and PsbV-ARG31. These residues thus might play a role in the attachment of PsbV, in an SQDG-mediated manner.

Furthermore, some of the bound lipids are near the PLQ exchange cavity, suggesting a role in steering the PLQ/PLQol exchange pathways. For instance, the SL1 binding site is located at the lumenal side of the PLQ exchange cavity, and might thereby influence the flux of PLQ and PLQol in and out of the cavity. Experiments show that SQDG deprivation results in changes around the Q_B binding site found inside the exchange cavity (35,41,42,83). The low mobility of the cocrystallized PG lipids 51 and 55, and 52 and 56, which are also located in and around the PLQ exchange cavity, supports a functional role, in agreement with the experiments of Itoh et al. (31).

Lipid exchange in the PLQ cavity

With the exception of the binding sites discussed above, we observed lipids in the exchange cavity to be rather mobile in general, and able to exchange with lipids from the bulk thylakoid membrane. The fluxes of lipids through the PLQ channels are of the order of a few tens of lipids ms^{-1} per monomer, similar to the exchange rates estimated for PLQ/PLQol in our previous study (65). The diffusion of lipids in and out of the cavity appears therefore coupled to the exchange of PLQ/PLQol in and out of PSII. For PLQ and PLQol, we previously showed that in addition to the known channels I and II, a third channel is used (65). Here we observed lipids to also exchange using channel III, in fact as the dominant route in addition to channel I. Channel II, being the narrowest channel, did not allow lipid flow in our simulations, but cannot be ruled out as an additional lipid channel as the opening and closing of the channels is a stochastic event (65). The high fluxes together with the existence of multiple channels suggest a much more open nature of the PLQ exchange cavity than the word "cavity" implies. PLQ exchange "bay" might be a more appropriate description. In this model, the PLQ exchange bay has a relatively open nature, where helices Cyt b 559$\alpha + \beta$ and PsbJ prevent the bay from being blocked by larger molecules and proteins. The promiscuous nature of the exchange may prevent high entropic energy barriers that are usually associated with ligand binding.

Limitations and outlook

Here we briefly discuss a number of limitations of our study that are important in the interpretation of our results, and provide an outlook for future studies.

The first limitation is the absence of lipid asymmetry in our simulations. In reality, lipid membranes typically have an asymmetric distribution of lipids between the two leaflets. In PSII, all cocrystallized MGDG lipids are located lumenally and all SQDG lipids stromally (8), indicative of the existence of membrane asymmetry. However, a structure of PSI with LHCI reveals cocrystallized DGDG and MGDG in the stromal leaflet (84). This result would make such an asymmetrical distribution of lipids over the two leaflets more unlikely. Due to lack of more quantitative information on lipid asymmetry in thylakoid membranes, we opted for a symmetric membrane model. The newly predicted binding sites of both MGDG and SQDG lipids are located on either side of the protein. Only when more conclusive data on lipid asymmetry become available can the relevance of these sites be reassessed.

The second limitation is that our simulations have not fully converged. By coarse-graining a system it becomes possible to sample much longer timescales, important to achieve statistically meaningful results on protein-lipid binding. Yet, even though we simulated for 87 μs, which is substantial for current practices, the simulation has not converged. The differences between the two monomers are the proof of this.

The third limitation pertains to the use of a CG model, implying a loss of detail as a tradeoff for the increased sampling speed. For instance, consider hydrogen bond

capabilities; although implicitly included in the parameterization of the various bead subtypes, their directionality is lost, potentially impacting the relative binding strength of the glycolipids. For an extensive discussion on limitations of the Martini model, we refer the reader to Marrink and Tieleman (85). To verify our CG model, one could simulate (parts) of the system on an all-atom level; for example, to study the interactions between some of the lipids and the protein in more detail. A complete all-atom model of PSII, including all cofactors and thylakoid lipids, could be constructed using efficient backmapping tools (86) and recently parameterized all-atom force fields of the cofactors and the thylakoid lipids (45,87–89).

Furthermore, one should be aware that in this study the PSII crystal structure of *Synechococcus vulcanus* was simulated, which means that care should be taken when interpreting these results for plants. The PSII cores of plants and cyanobacteria are very similar, but their antenna complexes are completely different. The cyanobacterial phycobilisome binds stromally to PSII, whereas the LHCII complexes in plants bind laterally to PSII, which is also the location of the lipid binding sites. One could therefore expect differences between the lipid binding sites in plant and cyanobacterial PSII. A second difference between plants and cyanobacteria that affects the lipid binding sites is the composition of their thylakoid membranes, which mainly differs in fatty acid composition.

Ultimately, our predictions require experimental validation. The experimental identification of lipid binding sites on membrane proteins is a rapidly growing area, thanks to an increasing number of techniques (90). Traditionally, x-ray techniques may reveal tightly bound, cocrystallized lipids, but advanced mutagenesis studies or chemical cross-linking techniques are also used to probe weaker bound lipids that are not washed away under the harsh crystallization conditions. New techniques such as the use of lipid nanodisks to isolate membrane proteins with their native lipid environment (91) and mass spectrometry (92) hold a lot of promise to further this development. Studies in which photosynthetic complexes are embedded in model membranes, that contain one or more of the thylakoid lipids, could further elucidate the role of specific lipids and their role in electron transport and supercomplex formation.

CONCLUSIONS

Taken together, we revealed specific lipid binding sites on a large multisubunit protein complex, PSII, embedded in a realistic lipid environment, the thylakoid membrane. Surprisingly, only MGDG and SQDG lipids form strong contacts with the PSII system, whereas DGDG and PG lipids are largely depleted. We find that charged amino acids and chlorophylls are important components of the lipid binding sites. Furthermore, we showed that the PLQ exchange cavity is much more dynamic than previously assumed, allowing the exchange of lipids along with the electron carriers. In general, our large-scale coarse-grain simulations open the way to improve our understanding of protein-lipid interactions in the complex setting of native membranes.

SUPPORTING MATERIAL

Supporting Materials and Methods, two figures, and four tables are available at http://www.biophysj.org/biophysj/supplemental/S0006-3495(17)31120-7.

AUTHOR CONTRIBUTIONS

F.J.V.E. and S.J.M. designed the research. F.J.V.E. and M.N.M. performed the research. All authors analyzed the data and wrote the manuscript.

ACKNOWLEDGMENTS

F.J.V.E. acknowledges funding from the Foundation of Fundamental Research of Matter (FOM), which is part of the Netherlands Organisation for Scientific Research (NWO). M.N.M acknowledges support from Project LISBOA-01-0145-FEDER-007660 funded by FEDER funds through COMPETE2020-POCI, and by national funds through Fundação para a Ciência e a Tecnologia. Both M.N.M. and P.W.J.M.F. acknowledge support from the Netherlands Organisation for Scientific Research (Veni, projects 722.013.010 and 722.015.005 respectively). S.J.M. acknowledges funding through an ERC Advanced Grant “COMP-MICR-CROW-MEM”. Furthermore, we thank the University of Groningen for access to the Peregrine Computing Cluster. This work was partly carried out on the Dutch national e-infrastructure with the support of SURF Cooperative.

REFERENCES

1. Blankenship, R. E. 2014. Molecular Mechanisms of Photosynthesis,, 2nd Ed. Wiley Blackwell, Hoboken, NJ.
2. Fromme, P., and I. Grotjohann. 2008. Structure of Photosystems I and II. *Results Probl. Cell Differ.* 45:33–72.
3. Brown, M. F. 2017. Soft matter in lipid-protein interactions. *Annu. Rev. Biophys.* 46:379–410.
4. Siegenthaler, P. A., and N. Murata. 2006. Lipids in Photosynthesis: Structure, Function and Genetics. Springer Science & Business Media, Berlin, Germany.
5. Sakurai, I., J.-R. Shen, …, H. Wada. 2006. Lipids in oxygen-evolving photosystem II complexes of cyanobacteria and higher plants. *J. Biochem.* 140:201–209.
6. Kansy, M., C. Wilhelm, and R. Goss. 2014. Influence of thylakoid membrane lipids on the structure and function of the plant photosystem II core complex. *Planta.* 240:781–796.
7. Jordan, P., P. Fromme, …, N. Krauss. 2001. Three-dimensional structure of cyanobacterial photosystem I at 2.5 Å resolution. *Nature.* 411:909–917.
8. Umena, Y., K. Kawakami, …, N. Kamiya. 2011. Crystal structure of oxygen-evolving photosystem II at a resolution of 1.9 Å. *Nature.* 473:55–60.
9. Guskov, A., J. Kern, …, W. Saenger. 2009. Cyanobacterial photosystem II at 2.9-A resolution and the role of quinones, lipids, channels and chloride. *Nat. Struct. Mol. Biol.* 16:334–342.
10. Shipley, G. G., J. P. Green, and B. W. Nichols. 1973. The phase behavior of monogalactosyl, digalactosyl, and sulphoquinovosyl diglycerides. *Biochim. Biophys. Acta.* 311:531–544.

11. Tilcock, C. P. 1986. Lipid polymorphism. *Chem. Phys. Lipids.* 40:109–125.

12. Garab, G., B. Ughy, and R. Goss. 2016. Role of MGDG and non-bilayer lipid phases in the structure and dynamics of chloroplast thylakoid membranes. *In* Lipids in Plant and Algae Development. Springer International, Cham, Switzerland, pp. 127–157.

13. Latowski, D., J. Kruk, …, K. Strzałka. 2002. Kinetics of violaxanthin de-epoxidation by violaxanthin de-epoxidase, a xanthophyll cycle enzyme, is regulated by membrane fluidity in model lipid bilayers. *Eur. J. Biochem.* 269:4656–4665.

14. Schaller, S., D. Latowski, …, R. Goss. 2010. The main thylakoid membrane lipid monogalactosyldiacylglycerol (MGDG) promotes the de-epoxidation of violaxanthin associated with the light-harvesting complex of photosystem II (LHCII). *Biochim. Biophys. Acta.* 1797:414–424.

15. Zhou, F., S. Liu, …, C. Yang. 2009. Effect of monogalactosyldiacylglycerol on the interaction between photosystem II core complex and its antenna complexes in liposomes of thylakoid lipids. *Photosynth. Res.* 99:185–193.

16. Schaller, S., D. Latowski, …, R. Goss. 2011. Regulation of LHCII aggregation by different thylakoid membrane lipids. *Biochim. Biophys. Acta.* 1807:326–335.

17. Jarvis, P., P. Dörmann, …, J. Chory. 2000. Galactolipid deficiency and abnormal chloroplast development in the Arabidopsis MGD synthase 1 mutant. *Proc. Natl. Acad. Sci. USA.* 97:8175–8179.

18. Krupa, Z., J. P. Williams, …, N. P. Huner. 1992. The role of Acyl lipids in reconstitution of lipid-depleted light-harvesting complex II from cold-hardened and nonhardened rye. *Plant Physiol.* 100:931–938.

19. Nussberger, S., K. Dörr, …, W. Kühlbrandt. 1993. Lipid-protein interactions in crystals of plant light-harvesting complex. *J. Mol. Biol.* 234:347–356.

20. Sakurai, I., N. Mizusawa, …, N. Sato. 2007. Digalactosyldiacylglycerol is required for stabilization of the oxygen-evolving complex in photosystem II. *Plant Physiol.* 145:1361–1370.

21. Gabashvili, I. S., A. Menikh, …, M. Fragata. 1998. Protein structure of photosystem II studied by FT-IR spectroscopy. Effect of digalactosyldiacylglycerol on the tyrosine side chain residues. *J. Mol. Struct.* 444:123–133.

22. Dörmann, P., S. Hoffmann-Benning, …, C. Benning. 1995. Isolation and characterization of an Arabidopsis mutant deficient in the thylakoid lipid digalactosyl diacylglycerol. *Plant Cell.* 7:1801–1810.

23. Härtel, H., H. Lokstein, …, C. Benning. 1998. Photosynthetic light utilization and xanthophyll cycle activity in the galactolipid deficient dgd1 mutant of *Arabidopsis thaliana*. *Plant Physiol. Biochem.* 36:407–417.

24. Ivanov, A. G., L. Hendrickson, …, N. P. Huner. 2006. Digalactosyldiacylglycerol deficiency impairs the capacity for photosynthetic intersystem electron transport and state transitions in *Arabidopsis thaliana* due to photosystem I acceptor-side limitations. *Plant Cell Physiol.* 47:1146–1157.

25. Guo, J., Z. Zhang, …, L. Zhang. 2005. Decreased stability of photosystem I in dgd1 mutant of *Arabidopsis thaliana*. *FEBS Lett.* 579:3619–3624.

26. Krumova, S. B., S. P. Laptenok, …, H. van Amerongen. 2010. Digalactosyl-diacylglycerol-deficiency lowers the thermal stability of thylakoid membranes. *Photosynth. Res.* 105:229–242.

27. Droppa, M., G. Horváth, …, T. Farkas. 1995. The role of phospholipids in regulating photosynthetic electron transport activities: treatment of thylakoids with phospholipase C. *Photosynth. Res.* 46:287–293.

28. Fragata, M., K. Strzałka, and E. K. Nénonéné. 1991. $MgCl_2$-induced reversal of oxygen evolution decay in photosystem II particles incubated with phosphatidylglycerol vesicles at high lipid/photosystem II ratio. *J. Photochem. Photobiol. B.* 11:329–342.

29. Jordan, B. R., W. S. Chow, and A. J. Baker. 1983. The role of phospholipids in the molecular organisation of pea chloroplast membranes. Effect of phospholipid depletion on photosynthetic activities. *Biochim. Biophys. Acta.* 725:77–86.

30. Gombos, Z., Z. Várkonyi, …, H. Wada. 2002. Phosphatidylglycerol requirement for the function of electron acceptor plastoquinone Q_B in the photosystem II reaction center. *Biochemistry.* 41:3796–3802.

31. Itoh, S., T. Kozuki, …, Z. Gombos. 2012. Two functional sites of phosphatidylglycerol for regulation of reaction of plastoquinone Q_B in photosystem II. *Biochim. Biophys. Acta.* 1817:287–297.

32. Kruse, O., B. Hankamer, …, J. Barber. 2000. Phosphatidylglycerol is involved in the dimerization of photosystem II. *J. Biol. Chem.* 275:6509–6514.

33. Sakurai, I., M. Hagio, …, H. Wada. 2003. Requirement of phosphatidylglycerol for maintenance of photosynthetic machinery. *Plant Physiol.* 133:1376–1384.

34. Sakurai, I., N. Mizusawa, …, H. Wada. 2007. Effects of the lack of phosphatidylglycerol on the donor side of photosystem II. *Plant Physiol.* 144:1336–1346.

35. Mizusawa, N., and H. Wada. 2012. The role of lipids in photosystem II. *Biochim. Biophys. Acta.* 1817:194–208.

36. Domonkos, I., P. Malec, …, Z. Gombos. 2004. Phosphatidylglycerol is essential for oligomerization of photosystem I reaction center. *Plant Physiol.* 134:1471–1478.

37. Sato, N., K. Suda, and M. Tsuzuki. 2004. Responsibility of phosphatidylglycerol for biogenesis of the PSI complex. *Biochim. Biophys. Acta.* 1658:235–243.

38. Härtel, H., P. Dormann, and C. Benning. 2000. DGD1-independent biosynthesis of extraplastidic galactolipids after phosphate deprivation in Arabidopsis. *Proc. Natl. Acad. Sci. USA.* 97:10649–10654.

39. Benning, C. 1998. Biosynthesis and function of the sulfolipid sulfoquinovosyl diacylglycerol. *Annu. Rev. Plant Physiol. Plant Mol. Biol.* 49:53–75.

40. Sato, N., M. Tsuzuki, …, A. Kawaguchi. 1995. Isolation and characterization of mutants affected in lipid metabolism of *Chlamydomonas reinhardtii*. *Eur. J. Biochem.* 230:987–993.

41. Minoda, A., N. Sato, …, M. Tsuzuki. 2002. Role of sulfoquinovosyl diacylglycerol for the maintenance of photosystem II in *Chlamydomonas reinhardtii*. *Eur. J. Biochem.* 269:2353–2358.

42. Minoda, A., K. Sonoike, …, M. Tsuzuki. 2003. Decrease in the efficiency of the electron donation to tyrosine Z of photosystem II in an SQDG-deficient mutant of Chlamydomonas. *FEBS Lett.* 553:109–112.

43. Gounaris, K., D. Whitford, and J. Barber. 1983. The effect of thylakoid lipids on an oxygen-evolving photosystem II preparation. *FEBS Lett.* 163:230–234.

44. Chain, R. K. 1985. Involvement of plastoquinone and lipids in electron transport reactions mediated by the cytochrome b_6-f complex isolated from spinach. *FEBS Lett.* 180:321–325.

45. Van Eerden, F. J., D. H. de Jong, …, S. J. Marrink. 2015. Characterization of thylakoid lipid membranes from cyanobacteria and higher plants by molecular dynamics simulations. *Biochim. Biophys. Acta.* 1848:1319–1330.

46. Ingólfsson, H. I., C. Arnarez, …, S. J. Marrink. 2016. Computational 'microscopy' of cellular membranes. *J. Cell Sci.* 129:257–268.

47. Mori, T., N. Miyashita, …, Y. Sugita. 2016. Molecular dynamics simulations of biological membranes and membrane proteins using enhanced conformational sampling algorithms. *Biochim. Biophys. Acta.* 1858 (7 Pt B):1635–1651.

48. Pluhackova, K., and R. A. Böckmann. 2015. Biomembranes in atomistic and coarse-grained simulations. *J. Phys. Condens. Matter.* 27:323103.

49. Aponte-Santamaría, C., R. Briones, …, B. L. de Groot. 2012. Molecular driving forces defining lipid positions around aquaporin-0. *Proc. Natl. Acad. Sci. USA.* 109:9887–9892.

50. Domański, J., S. J. Marrink, and L. V. Schäfer. 2012. Transmembrane helices can induce domain formation in crowded model membranes. *Biochim. Biophys. Acta.* 1818:984–994.

51. Grossfield, A., S. E. Feller, and M. C. Pitman. 2006. A role for direct interactions in the modulation of rhodopsin by omega-3 polyunsaturated lipids. *Proc. Natl. Acad. Sci. USA.* 103:4888–4893.

52. Koivuniemi, A., T. Vuorela, …, M. T. Hyvönen. 2012. Lipid exchange mechanism of the cholesteryl ester transfer protein clarified by atomistic and coarse-grained simulations. *PLOS Comput. Biol.* 8:e1002299.

53. Sengupta, D., and A. Chattopadhyay. 2015. Molecular dynamics simulations of GPCR-cholesterol interaction: an emerging paradigm. *Biochim. Biophys. Acta.* 1848:1775–1782.

54. Stansfeld, P. J., R. Hopkinson, …, M. S. P. Sansom. 2009. PIP_2-binding site in Kir channels: definition by multiscale biomolecular simulations. *Biochemistry.* 48:10926–10933.

55. Arnarez, C., J.-P. Mazat, …, X. Periole. 2013. Evidence for cardiolipin binding sites on the membrane-exposed surface of the cytochrome bc_1. *J. Am. Chem. Soc.* 135:3112–3120.

56. Hedger, G., S. L. Rouse, …, M. S. P. Sansom. 2016. Lipid-Loving ANTs: molecular simulations of cardiolipin interactions and the organization of the adenine nucleotide translocase in model mitochondrial membranes. *Biochemistry.* 55:6238–6249.

57. Vassiliev, S., T. Zaraiskaya, and D. Bruce. 2012. Exploring the energetics of water permeation in photosystem II by multiple steered molecular dynamics simulations. *Biochim. Biophys. Acta.* 1817:1671–1678.

58. Ogata, K., T. Yuki, …, S. Nakamura. 2013. All-atom molecular dynamics simulation of photosystem II embedded in thylakoid membrane. *J. Am. Chem. Soc.* 135:15670–15673.

59. Gabdulkhakov, A. G., V. G. Kljashtorny, and M. V. Dontsova. 2015. Molecular dynamics studies of pathways of water movement in cyanobacterial photosystem II. *Crystallogr. Rep.* 60:83–89.

60. Zhang, L., D.-A. Silva, …, X. Huang. 2014. Dynamic protein conformations preferentially drive energy transfer along the active chain of the Photosystem II reaction centre. *Nat. Commun.* 5:4170.

61. Chandler, D. E., J. Strümpfer, …, K. Schulten. 2014. Light harvesting by lamellar chromatophores in *Rhodospirillum photometricum*. *Biophys. J.* 106:2503–2510.

62. Ingólfsson, H. I., C. A. López, …, S. J. Marrink. 2014. The power of coarse graining in biomolecular simulations. *Wiley Interdiscip. Rev. Comput. Mol. Sci.* 4:225–248.

63. Wei, X., X. Su, …, Z. Liu. 2016. Structure of spinach photosystem II-LHCII supercomplex at 3.2 Å resolution. *Nature.* 534:69–74.

64. Van Eerden, F. J., T. van den Berg, …, S. J. Marrink. 2017. Molecular dynamics of photosystem II embedded in the thylakoid membrane. *J. Phys. Chem. B.* 121:3237–3249.

65. Van Eerden, F. J., M. N. Melo, …, S. J. Marrink. 2017. Exchange pathways of plastoquinone and plastoquinol in the photosystem II complex. *Nat. Commun.* 8:15214.

66. Wassenaar, T. A., H. I. Ingólfsson, …, S. J. Marrink. 2015. Computational lipidomics with Insane: a versatile tool for generating custom membranes for molecular simulations. *J. Chem. Theory Comput.* 11:2144–2155.

67. Marrink, S. J., H. J. Risselada, …, A. H. de Vries. 2007. The MARTINI force field: coarse grained model for biomolecular simulations. *J. Phys. Chem. B.* 111:7812–7824.

68. De Jong, D. H., G. Singh, …, S. J. Marrink. 2013. Improved parameters for the martini coarse-grained protein force field. *J. Chem. Theory Comput.* 9:687–697.

69. Periole, X., M. Cavalli, …, M. A. Ceruso. 2009. Combining an elastic network with a coarse-grained molecular force field: structure, dynamics, and intermolecular recognition. *J. Chem. Theory Comput.* 5:2531–2543.

70. López, C. A., Z. Sovova, …, S. J. Marrink. 2013. Martini force field parameters for glycolipids. *J. Chem. Theory Comput.* 9:1694–1708.

71. de Jong, D. H., S. Baoukina, …, S. J. Marrink. 2016. Martini straight: boosting performance using a shorter cutoff and GPUs. *Comput. Phys. Commun.* 199:1–7.

72. Bussi, G., D. Donadio, and M. Parrinello. 2007. Canonical sampling through velocity rescaling. *J. Chem. Phys.* 126:014101.

73. Berendsen, H. J. C., J. P. M. Postma, …, J. R. Haak. 1984. Molecular dynamics with coupling to an external bath. *J. Chem. Phys.* 81:3684–3690.

74. Castillo, N., L. Monticelli, …, D. P. Tieleman. 2013. Free energy of WALP23 dimer association in DMPC, DPPC, and DOPC bilayers. *Chem. Phys. Lipids.* 169:95–105.

75. Humphrey, W., A. Dalke, and K. Schulten. 1996. VMD: visual molecular dynamics. *J. Mol. Graph.* 14:33–38, 27–28.

76. Daura, X., K. Gademann, …, A. E. Mark. 1999. Peptide folding: when simulation meets experiment. *Angew. Chem. Int. Ed.* 38:236–240.

77. Loll, B., J. Kern, …, J. Biesiadka. 2005. Towards complete cofactor arrangement in the 3.0 Å resolution structure of photosystem II. *Nature.* 438:1040–1044.

78. Lins, R. D., C. S. Pereira, and P. H. Hünenberger. 2004. Trehalose-protein interaction in aqueous solution. *Proteins.* 55:177–186.

79. de Jong, D. H., C. A. López, and S. J. Marrink. 2013. Molecular view on protein sorting into liquid-ordered membrane domains mediated by gangliosides and lipid anchors. *Faraday Discuss.* 161:347–363, discussion 419–459.

80. Gu, R.-X., H. I. Ingólfsson, …, D. P. Tieleman. 2017. Ganglioside-lipid and ganglioside-protein interactions revealed by coarse-grained and atomistic molecular dynamics simulations. *J. Phys. Chem. B.* 121:3262–3275.

81. Liu, H., H. Zhang, …, R. E. Blankenship. 2013. Phycobilisomes supply excitations to both photosystems in a megacomplex in cyanobacteria. *Science.* 342:1104–1107.

82. Arnarez, C., S. J. Marrink, and X. Periole. 2016. Molecular mechanism of cardiolipin-mediated assembly of respiratory chain supercomplexes. *Chem. Sci.* 7:4435–4443.

83. Sato, N., M. Aoki, …, M. Tsuzuki. 2003. Involvement of sulfoquinovosyl diacylglycerol in the structural integrity and heat-tolerance of photosystem II. *Planta.* 217:245–251.

84. Qin, X., M. Suga, …, J.-R. Shen. 2015. Photosynthesis. Structural basis for energy transfer pathways in the plant PSI-LHCI supercomplex. *Science.* 348:989–995.

85. Marrink, S. J., and D. P. Tieleman. 2013. Perspective on the Martini model. *Chem. Soc. Rev.* 42:6801–6822.

86. Wassenaar, T. A., K. Pluhackova, …, D. P. Tieleman. 2014. Going backward: a flexible geometric approach to reverse transformation from coarse grained to atomistic models. *J. Chem. Theory Comput.* 10:676–690.

87. De Jong, D. H., N. Liguori, …, S. J. Marrink. 2015. Atomistic and coarse grain topologies for the cofactors associated with the photosystem II core complex. *J. Phys. Chem. B.* 119:7791–7803.

88. Debnath, A., S. Wiegand, …, C. Peter. 2015. Derivation of coarse-grained simulation models of chlorophyll molecules in lipid bilayers for applications in light harvesting systems. *Phys. Chem. Chem. Phys.* 17:22054–22063.

89. Manna, M., T. Róg, and I. Vattulainen. 2014. The challenges of understanding glycolipid functions: an open outlook based on molecular simulations. *Biochim. Biophys. Acta.* 1841:1130–1145.

90. Yeagle, P. L. 2014. Non-covalent binding of membrane lipids to membrane proteins. *Biochim. Biophys. Acta.* 1838:1548–1559.

91. Swainsbury, D. J. K., S. Scheidelaar, …, M. R. Jones. 2014. Bacterial reaction centers purified with styrene maleic acid copolymer retain native membrane functional properties and display enhanced stability. *Angew. Chem. Int. Ed. Engl.* 53:11803–11807.

92. Bechara, C., and C. V. Robinson. 2015. Different modes of lipid binding to membrane proteins probed by mass spectrometry. *J. Am. Chem. Soc.* 137:5240–5247.

Biophysical Journal, Volume *113*

Supplemental Information

Prediction of Thylakoid Lipid Binding Sites on Photosystem II

Floris J. Van Eerden, Manuel N. Melo, Pim W.J.M. Frederix, and Siewert J. Marrink

Supporting Material

Prediction of Thylakoid Lipid Binding Sites on Photosystem II

Floris J. Van Eerden[1], Manuel N. Melo[1,2], Pim W.J.M. Frederix[1], Siewert J. Marrink[1]

[1] Groningen Biomolecular Sciences and Biotechnology Institute & Zernike Institute for Advanced Materials, University of Groningen, Nijenborgh 4, 9747 AG Groningen, The Netherlands. [2] Instituto de Tecnologia Química e Biológica António Xavier, Universidade Nova de Lisboa, Av. da República, 2780-157 Oeiras, Portugal.

A description of every MGDG and SQDG binding site, two tables with details of the membrane-exposed binding sites (Table S1) and co-crystallized binding sites (Table S2), two tables with details on the co-crystallized lipids (Table S3, S4), and two figures with lipid residence time histograms (Fig S1) and lipid occupancies (Fig S2) for each binding site.

Detailed description of MGDG binding sites

Here we turn to a more detailed analysis of the 9 individual binding sites of MGDG (MS1-5 on the stromal side, and ML1-4 on the lumenal side), see Figures 3, 6, S1, S2 and Table S1.

MS1 is one of the two MGDG binding sites that are located at the dimer interface. The MS1 site tightly binds lipids with residence times reaching more than 80 µs, which is due to a strong coordination by CP47-CHL26, see Figure 6A. The MS1 site mostly binds a single lipid, and is occupied by MGDG 80% of the time.

MS2 is located close to the second PLQ exchange channel (1). This site is an intermediate strong binder, with residence times up to 48 µs. The binding site is partly made up by residues from the Cyt b 559β loop, which folds back into the membrane. The PsbX helix contributes to the site, but in one of the monomers this helix moves away during the simulation. A heme is also part of the binding site, but does not seem to interact very strongly with the lipids. MS2 also binds mostly a single lipid, and is occupied 89% of the time.

MS3 is located at the short side of PSII, between CP43 and PsbZ. It does not bind lipids very strongly, with a maximum residence time of 14 µs. However, it can bind multiple MGDGs at the same time. On average, two lipids are bound, and the occupancy level is correspondingly high (94% of the time at least one MGDG lipid is found at this site). CP43-CHL40 is the main residue contributing to the MS3 binding site.

MS4 is also located at the long side of PSII and is composed out of two loops of the CP43 subunit. It does not bind lipids very strongly, with a maximum residence time of 13 μs. Most lipids bind and unbind on a sub-microsecond time scale. Similar to MS3, the site can bind multiple MGDG lipids at the same time (on average 1.8 lipids), and has an occupancy level of 90%. In particular threonine is enriched in this binding site.

MS5 is located close to the dimer interface. It is an intermediate strong binder, with residence times up to 28 μs. The occupancy level is 76%, with typically a single MGDG lipid bound. MS5 is composed out of residues from D1 and PsbI.

ML1 is the second MGDG binding site that is located right at the dimer interface. Like the stromal site MS1, it is a strong binder with residence times up to 81 μs. In fact, most of the time the site has two or three MGDG lipids bound, see Figure 6B. The overall occupancy is 84%. CP47-CHL17 has a strong coordinating function. In the dimer interface residues from both monomers interact simultaneously with the individual lipids, thereby probably contributing to the long lipid residence times in this site.

The ML2 site is located at the CP47 subunit, relatively close to the dimer interface. It is not a strong binder, probably because the site is very exposed and does not shield the lipids from the bulk membrane. Nevertheless, the site is occupied 90% of the time, and binds on average 1.5 MGDG lipids.

ML3 is located close to PLQ exchange channel II (1). It binds on average 2.1 lipids and is occupied for 96% of the simulation. There is one odd lipid that resides for more than 70 μs at the site. At a certain point during the simulation, the residues D2-THR102 and Cyt b 559 α -PHE47 move away from each other, which results in the opening of a kind of 'cove' in the protein. Subsequently a lipid enters into the cove after which the two amino acids move back to their original positions and enclose the lipid. The 'cove' opens again for a few short moments during the simulation, but in the end the lipid escapes by diving with its headgroup underneath the enclosing residues.

ML4 is located next to CP43 and is composed entirely out of residues from CP43. It is very exposed to the membrane and it binds lipids only very weakly (sub-microsecond time scale). Like ML2 and ML3, the binding site can bind multiple lipids at the same time, resulting in a high occupancy level (93%).

Detailed description of SQDG binding sites

Here we present a more detailed analysis of the 4 individual binding sites of SQDG (SS1-3 and SL1), see Figures 3, S1, S2 and Table S1.

The stromal SQDG site SS1 is located in the corner between CP43 and PsbZ. The site is not a strong binder, and is occupied only about 75% of the time, mostly by a single lipid. The site contains two positively charged residues, Lys37 and Arg135. The site also contains a CHL, but the CHL does not appear to coordinate the SQDG lipids very strongly.

SS2 is located next to the CP43 subunit. It does bind lipids somewhat stronger than site SS1, with residence times up to 7 µs and an occupancy level of 86%. The CP43-Lys156 residue is likely the main residue responsible for attracting SQDG lipids to this site.

SS3 is located between CP43 and PsbI, it is formed by an elongated stretch of residues. Both residues from CP43 and PsbI contribute to the site. The site has an occupancy of 93%. One lipid has a residence time up to 84 µs. The oleoyl tail of this lipid penetrates into the protein between CP43 and PsbI. Here it is surrounded by and possibly interacts with CHL 33 and with the tails of CHL 10, 28, 32 and 34. These interactions might stabilize the lipid resulting in its high residence time.

At the lumenal side there is one large SQDG binding site (SL1) that can simultaneously bind two or three lipids, see Figure 6C. It is located between subunits Cyt b 559 α , Cyt b 559 β , PsbJ, PsbK and ycf12 at the same spot as the entry of the PLQ/plastoquinol (PLQol) exchange channel, which grants excess to the PLQ exchange cavity. The SL1 site is a strong binder, with residence times of 10s of µs. The occupancy level is 98%. The strong binding may be explained by the presence of Mg^{2+} and Ca^{2+} ions at the binding site. Interestingly, one SQDG lipid enters and subsequently leaves the PLQ exchange cavity during the simulation.

Supplementary Tables S1-S4

Table S1: Membrane lipid bindings sites in PSII with their composition, averaged over both monomers.[a]

Binding site	Amino acids composing the binding site
	MGDG
	Stromal
MS1	CP47-LEU122, CP47-PHE123, CP47-ARG124, CP47-ASP125, CP47-ALA132, CP47-LEU133, CP47-ASP134, CP47-MET138, CP47-HIS142, CP47-CHL26, CP47-CHL27, PsbH-LEU7, PsbH-LEU11
MS2	D2-TRP21, D2-LEU22, D2-LYS23, D2-ARG24, D2-ASP25, D2-ARG26, D2-PHE27, D2-VAL28, D2-PHE29, D2-VAL30, Cyt b 559 α -TYR19, Cyt b 559 α -ILE22, Cyt b 559 α -HIS23, Cyt b 559 α -THR26, Cyt b 559 β -SER3, Cyt b 559 β -ASN4, Cyt b 559 β -VAL18, Cyt b 559 β -HEM641
MS3	CP43-CHL40
MS4	CP43-ASN155, CP43-LYS156, CP43-THR 158, CP43-THR159, CP43-HIS251, CP43-ILE252, CP43-LEU253 CP43-THR254, CP43-THR255, CP43-PRO256, CP43-PHE257
MS5	D1-ASN12, D1-TRP14, D1-GLU15, D1-CYS18, D1-TRP32, PsbI-GLY22, PsbI-PHE23, PsbI-SER25, PsbI-GLY26, PsbI-ASP27, PsbI-ALA29, PsbI-ARG30
	Lumenal
ML1[b]	D1-SER101*, D1-LEU102*, D1-ASP103*, CP47-TRP75, CP47-ASP87, CP47-PRO88, CP47-GLY89, CP47-PHE90, CP47-TRP91, CP47-CHL17, PsbO-LYS69*
ML2	CP47-ALA182, CP47-PRO183, CP47-GLU184, CP47-TRP185, CP47-GLY186
ML3	D2-ASP100, D2-PHE101, D2-THR102, D2-ARG103, Cyt b 559 α -PHE47
ML4	CP43-ASN201, CP43-PRO202, CP43-THR203, CP43-LEU204, CP43-ASP205
	SQDG
	Stromal
SS1	CP43-TRP33, CP43-LYS37, CP43-TYR131, CP43-ARG135, CP43-CHL40 PsbZ-TYR27, PsbZ-TRP33
SS2	CP43-SER144, CP43-SER145, CP43-PHE146, CP43-TYR149, CP43-LYS156, CP43-THR159
SS3	CP43-THR254, CP43-THR255, CP43-PRO256, CP43-PHE257, CP43-GLY258, CP43-TRP259, CP43-ARG261, CP43-ARG262, PsbI-SER25, PsbI-GLY26, PsbI-LYS35
	Lumenal
SL1	Cyt b 559 α -SER39, Cyt b 559 α -THR40, Cyt b 559 α -GLY41, Cyt b 559 β -GLN41, Cyt b 559 β -PHE42, Cyt b 559 β -ILE43, Cyt b 559 β -GLN44, Cyt b 559 β -ARG45, Cyt b 559 β -Ca^{2+}796, PsbJ-TYR30, PsbJ-ALA34, PsbJ-GLY35, PsbJ-LEU36, PsbJ-MG^{2+}771, ycf12-VAL18

[a] Binding sites are labelled as follow: first letter is the first letter of the lipid head group name (M for MGDG, S for SQDG), second letter indicates location, S for stromal and L for lumenal, followed by a sequential number. Positively charged residues are in blue and negatively charged residues in red.
*[b] The ML1 site is composed out of residues from both monomers, here an * behind a residue indicates that the residue is not part of the monomer to which the binding site is assigned, but belongs to the other monomer.*

Table S2: Co-crystalized lipids and their mobility.[a]

Nr	Headgroup	Mobility	Remarks
			Lumenal lipids
5	DGDG	None	
6	DGDG	None	
11	PG	Limited	
12	PG	Large	
13	SQDG	Limited	
14	SQDG	None	
21	SQDG	Limited	
22	SQDG	Large	An extra SQDG lipid enters the PLQ exchange cavity
23	SQDG	Limited	
24	SQDG	Large	An extra SQDG lipid enters the PLQ exchange cavity
27	SQDG	Limited	
28	SQDG	Limited	
29	SQDG	Limited	
30	SQDG	Limited	
45	SQDG	None	
46	SQDG	Escape	Escapes after diffusion of PsbX
47	PG	Limited	
48	PG	Limited	
49	PG	None	
50	PG	None	
51	PG	Limited	
52	PG	None	
55	PG	Limited	
56	PG	Limited	
59	PG	Limited	
60	PG	None	
61	MGDG	Escape	
62	MGDG	Escape	
			Stromal lipids
1	DGDG	None	
2	DGDG	None	
3	DGDG	None	
4	DGDG	None	
7	DGDG	Exchange	Lipid 7 & 20 are basically at the same spot, they both escape, but only one DGDG lipid returns
8	DGDG	Escape	
9	DGDG	Escape	No lipid density visible, so therefore not shown in Figure 3
10	DGDG	Escape	No lipid density visible, so therefore not shown in Figure 3
17	DGDG	None	
18	DGDG	Escape	The escape is a result of the movement of PsbX, which moves away and opens up the binding site. No lipid density visible in Figure 3.
19	DGDG	Escape	
20	DGDG	Exchange	Lipid 7 & 20 are basically at the same spot, they both escape, but only one DGDG lipid replaces them
25	MGDG	Limited	
26	MGDG	Limited	
31	MGDG	None	
32	MGDG	None	
33	DGDG	None	
34	DGDG	None	
35	DGDG	Exchange	Leaves the PLQ exchange cavity and gets replaced by another DGDG lipid
36	DGDG	Limited	
37	DGDG	Limited	
38	DGDG	None	
39	MGDG	Exchange	Leaves the PLQ exchange cavity and gets replaced by two MGDG lipids
40	MGDG	Limited	
41	MGDG	Exchange	Gets replaced by weak binding lipids including MGDG
42	MGDG	Exchange	Gets replaced by weak binding lipids including MGDG
43	DGDG	Exchange	Gets replaced by a strong binding MGDG lipid.
44	DGDG	Escape	Escape seems to be unrelated with movement of PsbX. No density visible in Figure 3.
53	MGDG	Limited	
54	MGDG	Limited	A second MGDG enters the PLQ exchange cavity
57	DGDG	None	
58	DGDG	None	

[a] *Numbering as in Fig. 3. The lipid mobility has been classified as 'none' or 'limited' for lipids that show no or very limited movement, 'large' for lipids that show a significant displacement and 'escape' for lipids that leave the binding site. In case an escaped lipid is replaced by another lipid, it is classified as 'exchange'. Note that nr. 15 and 16 are not included in this list, because they are beta carotenes.*

Table S3: Overview of the number of co-crystallized lipids per mobility category.[a]

Lipid	Total Number	None	Limited	Large	Exchange	Escape
				Total		
PG	12	4 (33%)	7 (58%)	1 (8%)	-	-
DGDG	24	12 (50%)	2 (8%)	-	4 (17%)	6 (25%)
MGDG	12	2 (17%)	5 (42%)	-	3 (25%)	2 (17%)
SQDG	12	2 (17%)	7 (58%)	2 (17%)	-	1 (8%)
				Stromal		
PG	12	4 (33%)	7 (58%)	1 (8%)	-	-
DGDG	2	2 (100%)	-	-	-	-
MGDG	2	-	-	-	-	2 (100%)
SQDG	12	2 (17%)	7 (58%)	2 (17%)	-	1 (8%)
				Lumenal		
PG	-	-	-	-	-	-
DGDG	22	10 (45%)	2 (9%)	-	4 (18%)	6 (27%)
MGDG	10	2 (20%)	5 (50%)	-	3 (30%)	-
SQDG	-	-	-	-	-	-

[a]*Numbers are shown for each leaflet as well as totals for the complete bilayer. For each lipid, the percentages for the mobility types are shown between brackets.*

Table S4: Lipid diffusion of co-crystalized and membrane lipids in and out of the PLQ exchange cavity.[a]

Monomer	PG		DGDG		MGDG		SQDG	
	in	out	in	out	in	out	in	out
				Stromal				
left	-	-	-	-	1 (I)	1 (I)	1 (I)	-
right	-	-	-	-	1 (III)	1 (III)	-	-
				Lumenal				
left	-	-	-	-	1 (I)	-	-	-
right	-	-	-	1 (III)	4 (III)	3 (III)	1 (III)	1 (III)

[a] *The number of lipids that enter and leave the PLQ exchange cavity is discriminated for each leaflet and monomer. The channel used by the lipids is shown between parentheses. Channel I refers to the PLQ channel as described in (2). Channel III is located between PsbJ on one side and between PsbK and ycf12 on the other side, and opens when PsbJ moves towards Cyt b 559α (3). 'Left' refers to the monomer in Figure 3 where the bindings sites are marked with an '*' and 'right' to the monomer where the binding sites are marked with a '#'.*

Supplementary Figures S1-S2

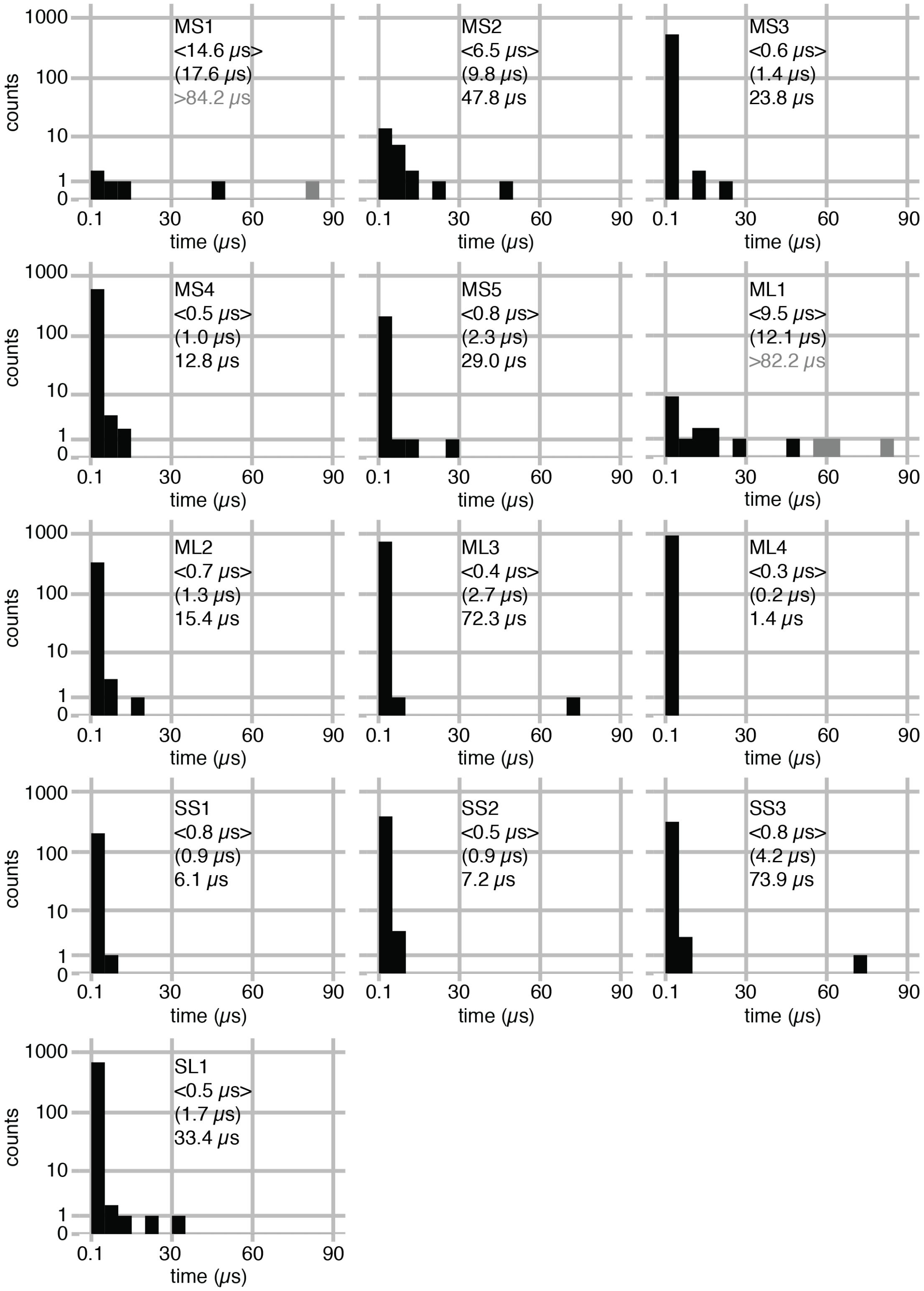

Figure S1: Lipid residence times in the different lipid binding sites. *For each binding site there is a histogram, with on the x-axis the residence time and on the y-axis the number of binding events (note that a single lipid could bind multiple times). The inset in each histogram shows the name of the binding site, the average residence*

time, its standard deviation and the longest residence time for that site. Residence times of lipids that are still bound at the end of the simulation and that were bound for at least 50 µs are coloured grey and not included in the calculation of the average residence time. If this lipid has the highest residence time of its site, the longest residence time in the inset represents a lower limit and is coloured grey as well. Note that a lower cut-off of a residence time of 100 ns was applied. The graphs and averages are calculated using the combined data of the two monomers.

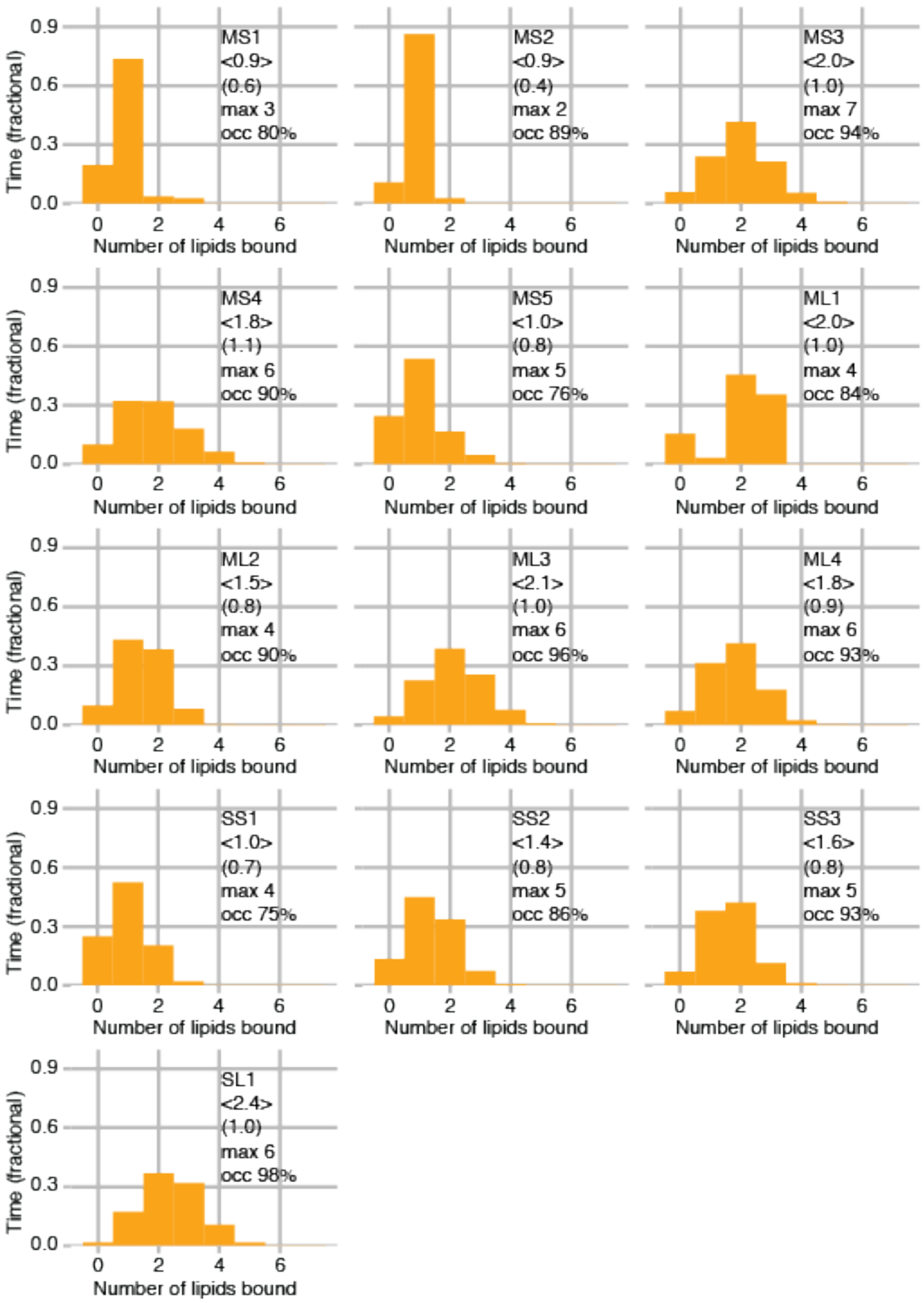

Figure S2: Lipid occupancy per binding site. *For each binding site there is a histogram, with on the x-axis the number of lipids bound and on the y-axis the fractional simulation time. The inset in each histogram shows the name of the binding site, the average number of lipids bound, its standard deviation, the maximum number of lipids bound for that site and the occupancy percentage. The data for each binding site represents the average over the two monomers, except for the maximum numbers of lipids bound, which is the maximum over the two monomers. Note that all lipids that bind were included, i.e., no lower cut-off of a 100 ns residence time was used.*

Supplemental References

1. Guskov, A., J. Kern, A. Gabdulkhakov, M. Broser, A. Zouni, and W. Saenger. 2009. Cyanobacterial Photosystem II at 2.9-Å Resolution and the Role of Quinones, Lipids, Channels and Chloride. Nat. Struct. Mol. Biol. 16: 334–342.

2. Loll, B., J. Kern, W. Saenger, A. Zouni, and J. Biesiadka. 2005. Towards complete cofactor arrangement in the 3.0 Å resolution structure of photosystem II. Nature. 438: 1040–1044.

3. van Eerden, F.J., M.N. Melo, P.W.J.M. Frederix, X. Periole, and S.J. Marrink. 2017. Exchange pathways of plastoquinone and plastoquinol in the photosystem II complex. Nat. Commun. 8: 15214.
